# Stochastic Resonance in Organic Electronic Devices

**DOI:** 10.3390/polym14040747

**Published:** 2022-02-15

**Authors:** Yoshiharu Suzuki, Naoki Asakawa

**Affiliations:** Molecular Science Division, Gunma University, Kiryu 376-8515, Gunma, Japan; t162a005@gunma-u.ac.jp

**Keywords:** polymer semiconductor, organic electronic device, polyalkylthiophene, stochastic resonance, noise-driven phenomenon, internal noise, biomimicry, bioinspiration, stochastic computing

## Abstract

Stochastic Resonance (SR) is a phenomenon in which noise improves the performance of a system. With the addition of noise, a weak input signal to a nonlinear system, which may exceed its threshold, is transformed into an output signal. In the other words, noise-driven signal transfer is achieved. SR has been observed in nonlinear response systems, such as biological and artificial systems, and this review will focus mainly on examples of previous studies of mathematical models and experimental realization of SR using poly(hexylthiophene)-based organic field-effect transistors (OFETs). This phenomenon may contribute to signal processing with low energy consumption. However, the generation of SR requires a noise source. Therefore, the focus is on OFETs using materials such as organic materials with unstable electrical properties and critical elements due to unidirectional signal transmission, such as neural synapses. It has been reported that SR can be observed in OFETs by application of external noise. However, SR does not occur under conditions where the input signal exceeds the OFET threshold without external noise. Here, we present an example of a study that analyzes the behavior of SR in OFET systems and explain how SR can be made observable. At the same time, the role of internal noise in OFETs will be explained.

## 1. Introduction

Since the 1990s, digital technology has developed rapidly and is now an important technology that supports society. The semiconductor devices are responsible for the digital processing, and most of them today are composed of complementary metal oxide semiconductor (MOS) [1]. Advances in CMOS miniaturization and integration technology have greatly improved the performance of digital computers. The metal oxide semiconductor field effect transistor (MOSFET) used in CMOS is an electronic device consisting of three terminals (gate, drain and source), and the current between the source and drain can be switched by an electrical signal applied to the gate. When the MOSFET is miniaturized, the drive voltage is lowered and the current flowing is reduced, thus suppressing power consumption. In addition, the amount of charge required for switching can be reduced, which leads to higher device speed. Furthremore, with miniaturization, it became possible to integrate more elements per unit area. In 1971, Intel developed the world’s first single-chip macroprocessor, the Intel 4004, which consisted of 2300 transistors and had a clock frequency of 740 kHz [2]. From there, the company released various types of processors and showed the progress of integration. From mid 2000s, the company switched their strategy from a single-core processor to multi-core processors, and it has continued till now. The rate of increase in the integration of such semiconductors is said to be about double in two years [3,4]. This law is called Moore’s Law, and the miniaturization of semiconductor devices has been progressing according to this law until now [5].

However, miniaturization according to Moore’s Law is considered to be broken [4]. In recent years, the process rule has been sub-10nm, and is about to reach the size of the atomic level, because there is a growing need to create devices that are so tiny that they are physically difficult to realize. There are also problems that have emerged as miniaturization has progressed. Normally, current should not flow through the insulating film, but if it is made too thin, a leakage current is generated. It also happens that the subthreshold current increases [6]. This causes the problem that the power consumption becomes large. In addition, the high level of integration combined with the high heat generation is also a challenge [5]. In other words, it can be said that the semiconductor devices that support digital information processing systems, which have achieved great development, are reaching the limit of their usefulness only with the strategy of miniaturization and integration as in the past.

On the other hand, the demand for semiconductor devices that are responsible for digital processing has been increasing more and more. As computers and cellular phones become more and more popular, the IoT [7], which connects a variety of things to the Internet to enable communication, is becoming more and more popular. Furthermore, the movement to utilize the big data obtained by using them is also accelerating [8]. Since these use digital processing systems, it is not hard to imagine that more semiconductor device elements are needed. The increase in the number of situations in which digital information processing is used means that more power is consumed by semiconductor devices, and the amount of energy used for information processing is expected to increase enormously. Therefore, it can be said that in order to develop information technology, there is a need to build a novel information processing system.

In 2012, Google was able to recognize cats through deep learning with unsupervised learning [9]. Also in 2012, AlexNex, which uses deep learning, won the ImageNet Large-Scale Visual Recognition Challenge (ILSVRC), an image recognition contest [9]. These led to a lot of attention to deep learning.

Deep learning uses an artificial neural network inspired by the biological brain [10]. When the sum of the input signals from the various synapses exceeds the threshold of the neuron, the neuron produces a large output. Each synapse has its own weighting. Deep learning is one that incorporates these neuronal features and has many hidden layers between the input and output layers. In other words, by incorporating some of the information processing mechanisms of living organisms, new functions have been obtained.

But even in this kind of machine learning, the foundation is digital technology. The hardware used for machine learning are digital-based semiconductors such as central processing unit (CPU), graphics processing unit (GPU), field-programmable gate array (FPGA), and application specific integrated circuit (ASIC) [11]. In order to perform machine learning, hardware with high processing power is required, and the performance of semiconductor devices must be improved. These digital-based systems are not necessarily optimized to mimic biological neural systems.

On the other hand, in addition to the above digital technologies, neuromorphic devices that implement a new information processing mechanism called cognitive computing [12], which mimics the advanced information processing such as cognition and decision making performed by humans, have been of attraction in recent years. The research field of neuromorphic devices can be divided into two major categories: one is based on very large scale integrated circuit (VLSI) technology, and the other stream is based on mathematical models using nonlinear physics and theoretical neuroscience.

In the former, the SyNAPSE project by DARPA and IBM, which started in 2008, is mainly based on VLSI technology, and researchers in materials science are also participating in the project [13]. In 2005, a project included the University of Manchester’s SpiNNaker [14], which later evolved into the European Union’s Human Brain Project [15], and in 2011, Heidelberg University’s BrainScaleS [16], which is an analog technology. The EU’s Human Brain Project, launched in 2013, is a holistic project on brain function, structure, use and medicine [15]. Later, in the 2014, IBM announced the TruNorth chip, which gathered a lot of attention [17]. After that, IBM has developed several neuromorphic projects that includes crossbar arrays with memristors [18]. In addition, Intel has announced a processor called Loihi [19] in 2017. The above is the flow of research and development mainly based on VLSI technology.

The latter approach to neuromorphic devices has long been a major trend based on chaotic dynamics included in nonlinear physics, and is known as the neuro-computer [20,21]. Also, amoeba was used as a key element for bio-inspired information processing [22,23]. Furthermore, over the recent decade, biomimetic signal/information processing material-based devices has been of attraction. Particularly, inorganic oxide semiconductors [24] and organic semiconductors [25,26,27,28] as well as numerical simulations and analog electric circuits [29,30] were used for fluctuation-driven signal transmission devices. Independently, in the field of IoT, especially, organic neuromorphic devices [31,32,33] have been of attention due to the expectation of organic electronic devices as biological IoT devices [34,35]. Under such circumstances, this review focuses on the stochastic resonance phenomenon, which is the basis of stochastic computing in neuronal systems [36], among organic neuromorphic devices, and reviews the current status and problems of organic devices in stochastic signal processing and information processing.

## 2. Information Processing in Biological Systems Using Noise

### 2.1. Stochastic Resonance Phenomena in Biological Organisms

**Figure 1:**
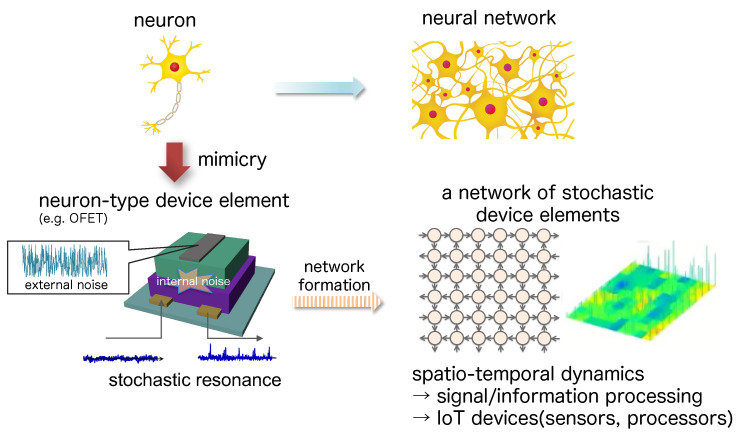
Neuron-type device elements, e.g., organic field-effect transistors (OFETs), become noise-driven signal transduction elements, i.e., stochastic resonance device elements. The basic unit based on such an OFET is made into a network device. The spatio-temporal patterns created by the output signals of each element in the network will be used in intelligent IoT devices such as sensors and processors.

A nervous system, which is thought of as an information processing system, is small in its power consumption compared to a digital computer [37]. This is partly because external and/or internal noise is used in the processing [38]. The most studied mechanism by which noise can be exploited is the stochastic resonance phenomenon [39,40]. Stochastic resonance is a phenomenon in which the signal transmission performance is improved by applying noise. For example, if a weak subthreshold input signal enters into a nonlinearly responding system which has a threshold, the system produces output signal with zero amplitude, but by adding noise at the same time as the input signal, the system may be able to produce an output that corresponds to the input signal (Figure 1). Such a phenomenon is called stochastic resonance.

This phenomenon has been experimentally confirmed in the sensory systems of living organisms. For example, Douglass et al. applied weak signal pulses to mechanoreceptor cells in the tail of the crayfish and observed the spiking activity of the cells when the pulses were applied [41]. When noise was applied simultaneously with weak signal pulses, the signal-to-noise ratio (SNR) of the output pulses increased, confirming the occurrence of stochastic resonance phenomena in living organisms. Also, crayfish are thought to sense predators by stochastic resonance using aquapressure waves [42]. Furthermore, SR phenomenon was observed in planktonic predation of paddlefish [43]. It is thought that SR may also be used for sensing natural enemies of crickets [44]. Other stochastic resonance experiments have also been performed in rats [45,46].

Experiments on stochastic resonance have also been conducted on human subjects. It was confirmed that visual [47], contact sensation [48] and balance control ability [49] were improved by noise. From the results described so far, it is possible that by using noise that exists outside a system, the system detects weak signals that cannot normally be detected or recognized.

### 2.2. Relationship between Noise-Induced Phenomena and Biological Functions

The behavior of neurons, which are responsible for information processing in living organisms, has been reproduced by mathematical models. Studies using neuronal models have shown that stochastic resonance also occurs in excitatory systems, and this has been confirmed under a variety of conditions. Collins et al. used the FitzHugh-Nagumo (FHN) neuron model in the spatially extended form of a parallel network with output summation, where independent noise is applied to individual neurons [50]. The results show that as the number of parallelization is increased, the effect of stochastic resonance is enhanced and the signal transmission performance once improved remains high even when the noise intensity is further increased. This model is called the Collins model. Parallelization, as in the Collins model, means that the reliability of signal transmission by stochastic resonance can be increased and the range of noise intensity that can be used to improve the signal transmission capability can be widened.

**Figure 2:**
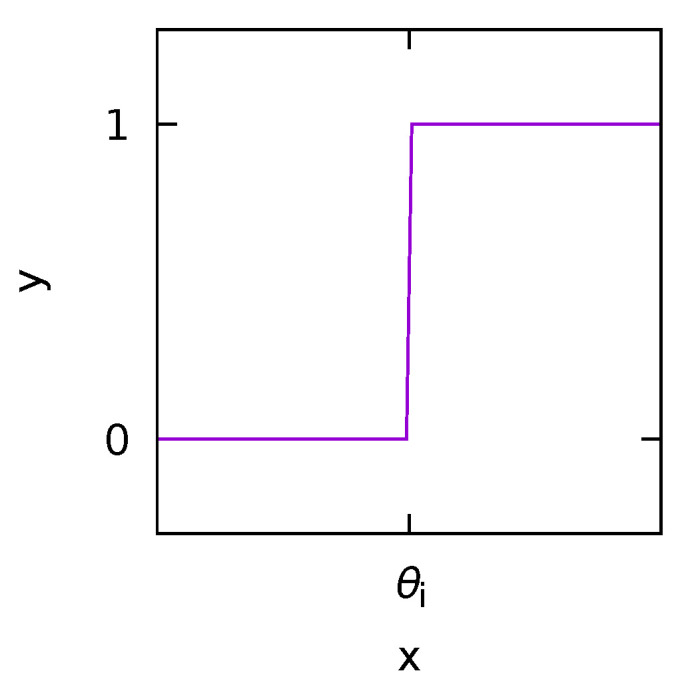
Simple threshold system using heaviside function modeled by y=θ(x), where *x*: input, *y*: output, and $\theta_{i}$: threshold.

Furthermore, for simpler threshold systems (Figure 2), where heaviside function modeled by y=θ(x) (*x*: input, *y*: output, $\theta_{i}$: threshold), it is known that stochastic resonance is observed even under the condition that the input signal is noiseless and above the threshold in the parallelized model.

This is called suprathreshold stochastic resonance [51,52,53]. Under conditions where the input signal is above a threshold, in many cases no stochastic resonance is seen when noise is applied [54,55]. However, in the above system, noise can improve signal transmission even when the input signal is large, which means that stochastic resonance is not only applicable to weak signals.

It is also known that stochastic resonance occurs when noise is introduced at the same time when a subthreshold signal consisting of multiple frequency components is input. Increasing the noise intensity applied together with the summed signal of two different frequencies $f_{1}$ and $f_{2}$, one can obtain the beat frequency ($f_{1}-f_{2}$) component, which is conducive at optimal noise intensity [56]. However, the amount of signal enhancement by stochastic resonance does not depend only on the beat frequency, but is also affected by the respective input frequencies $f_{1}$ and $f_{2}$.

**Figure 3:**
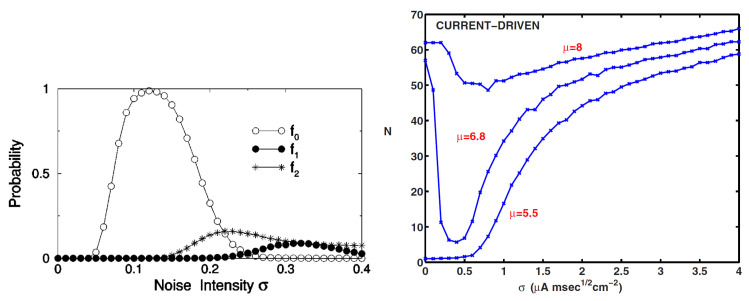
(**left**) Example of ghost stochastic resonance. The probability of observing $f_{0},f_{1}$ and $f_{2}$ spike frequencies for the variance of the noise σ given an input consisting of two frequencies ($f_{1}$, $f_{2}$). The largest resonance is observed in the weak wave $f_{0}$, which has no input ($f_{0}=1$ Hz, $f_{1}=2$ Hz, $f_{2}=3$ Hz): [57] adapted with permission from *Physical Review E* **2002**, *65*, 050902. Copyright 2002 American Physical Society. (**right**) Example of inverse stochastic resonance. The average number of spikes *N*(100 trials) with respect to a noise level σ in the Hodgkin-Huxley neuron model. The results are shown for three different average current intensities (average input current density μ) of input current: [59] adapted with permission from *Physical Review E* **2009**, *80*, 031907. Copyright 2009 American Physical Society.

In addition, there are studies that include noise at the same time as the signal consisting of harmonics [57,58]. When the multiple frequency components of the input signal are $kf_{0}$, $\left(k+1\right)f_{0}$, ⋯, $\left(k+n\right)f_{0}$ (k>0), the fundamental frequency component $f_{0}$ appears when the noise intensity is increased (Figure 3 (left)).

This means that a frequency component that does not exist in the input signal has appeared due to the application of noise. This phenomenon is called ghost stochastic resonance. Ghost stochastic resonance occurs even when the input signal is an anharmonic signal. If the input signal components are $f_{1}=kf_{0}+\Delta f$, $f_{2}=\left(k+1\right)f_{0}+\Delta f$, ⋯, if noise is applied when $f_{n}=\left(k+n\right)f_{0}+\Delta f$, the $f_{r}=f_{0}+\Delta f/\left(k+1/2\right)$ component appears at the output. This phenomenon has also been confirmed in experiments on living organisms [60,61]. It is said that this may be a mechanism that allows the auditory periphery to detect complex pitches of sound. Thus, the stochastic resonance phenomenon may play a role in supplementing the information to be transmitted.

The basic principle of stochastic resonance phenomena is that an input signal that cannot exceed a threshold value can be made to exceed it with the help of noise. In other words, in a neuron, noise is used to fire a neuron that cannot fire because the signal it is receiving is below the threshold. This means that noise has an excitatory (activating) effect. On the other hand, the effect of noise-induced suppressiveness has also been confirmed. For the Hodgkin-Huxley (HH) neuron model, it was reported that the firing frequency of neurons is minimized at the optimal noise intensity when noise is applied to the HH neuron [59,62,63]. In ordinary stochastic resonance phenomena, when the horizontal axis is intensity of applied noise and the vertical axis is signal transmission performance such as signal-to-noise ratio or mutual correlation between input and output signals, a bell-shaped curve can be seen. However, in this research, when the applied noise intensity is taken on the horizontal axis and the firing frequency on the vertical axis, the curve showed an inverted bell-shaped curve. Since this behavior is an upside-down reversal of the bell shape in many stochastic resonance phenomena, the phenomenon is called inverse stochastic resonance(Figure 3 (right)). As an actual biological system, this phenomenon has been confirmed in squid axons [64].

It has been reported that stochastic resonance is also effective in networks with a large number of excitatory and inhibitory neurons [65]. In this research, a leaky-integrate-and-fire (LIF) neuron model was used. The distribution of coupling strength between each neuron is long-tailed and a large number of weak connections and a small number of strong connections are present. If we focus on a single neuron, it was confirmed that the signal from many weak connections creates noise and the signal of a few strong connections was strengthen. Similarly, in neural networks, signals are transmitted through sparse, strong connections and assisted by input from a number of other weak bonds. This suggests that stochastic resonance phenomena contribute to signal transmission at the network level and that a large number of weak synaptic connections could be exploited for stochastic resonance.

The stochastic resonance described so far is a phenomenon that occurs when a signal is input to a nonlinear response system. On the other hand, there is a phenomenon called coherence resonance (CR), in which the application of noise causes the system to exhibit a quasi-periodic response even in the absence of an input signal. CR has been confirmed in excitable systems including HH neuron model [66,67], FHN neuron model [68,69,70], and Izhikevich neuron model [71]. When noise is applied to a neuron under the condition of little or no firing, the frequency of firing increases, and at the optimal noise intensity, the degree of synchronization increases and coherence resonance is emerged.

As described above, researches using numerical simulations with various mathematical models have led to the scenario that neurons can make effective use of internal and external noise. This suggests that stochastic resonance and other noise-induced phenomena may contribute to signal detection and information processing in nervous systems. However, it is still unclear whether stochastic resonance phenomena are used in the information processing in the actual expression of biological functions [72]. In most stochastic resonance experiments using biological systems, the effect of noise is investigated by applying an external noisy stimulus and studying the response to the stimulus. But living organisms, even without the addition of external noise, are able to perceive sufficiently large stimuli from the sensory organs to recognize, judge, and act flexibly. In other words, the nervous system seems to functions as maintained without external noise. Therefore, it can be said that the experimentally confirmed stochastic resonance in living organisms is merely a confirmation of the response of the sensory nerves, treating them as a nonlinear response system. From the above disccussion, if stochastic resonance phenomena are utilized in brain functions, it could be more reasonable to think that noise from inside the system is utilized. However, in order to check whether the stochastic resonance phenomenon is caused by the internal noise in the living organisms, we must be able to control the internal noise intensity when the organism is processing information. As long as there is no way to control the internal noise in the body, it is not clear whether stochastic resonance contributes to biological information processing explicitly or not.

In the following sections in this review, we try to describe various examples for application of noise in electronic devices (Section 3), and particularly in organic electronic devices (Section 4). Then, we shed light on noise-driven signal processing using stochastic resonance menomenon in OFETs (Section 5) and experimental realization of stochastic resonance phenomenon with applying external noise to an OFET system (Section 6). Furthemore, investigation of the effect of noise in the physical system, such as internal noise in OFETs, is also reviewed (Section 7). Finally, the effect of internal noise on the stochastic resonance is also analyzed numerically (Section 8).

## 3. Noise Utilization in Electronic Devices

### 3.1. Neuron-Type Device

In order to implement mechanisms of bioinspired sensing and information processing, the development of computers with brain-like mechanisms has been attempted [73,74]. Although artificial synapses have been created using CMOS technology, [75,76] device structures to represent active potential for these system are complicated and their energy consumptions are large as well as the physical size of the system. Therefore, proposed so far have been silicon based transistors with floating gate structure [77,78], memristors [79,80,81,82], ferroelectric devices [83,84], phase transition memories [85,86,87], and field effect transistors (FETs) [87,88,89,90,91]. Recently, highly interconnected, low-energy-driven synaptic devices have also been reported [75], and various inorganic synaptic devices [92,93,94,95,96,97] and network devices [98,99] have been developed.

In addition, organic synaptic devices are promising [31], because flexibility and stretchability inherent in organic materials make them ideal for neuromorphic systems that require complex information processing but have flexibility (e.g., for the fabrication of flexible intelligent systems [100,101]). In systems that integrate a large number of sensors and electronic devices, a neural network-like architecture is considered to be more suitable due to the need to process a large number of input signals [100,102].

Synaptic plasticity is thought to be important in the processing of encoding, storing, and reinforcing information as it is done in nervous systems [103]. Therefore, neuromorphic devices have been developed [104] so as to reproduce the Hebb rule [105], which explains the principle of synaptic plasticity, and the spike timing dependent plasticity [106].

On the other hand, electronic devices that mimic the characteristics of neurons in terms of stochastic response have been proposed. For example, a neuron-like molecular network has been reported in which noisy spikes are generated by single-walled carbon nanotubes (SWNTs) and polyoxometalate molecules [107]. There are also devices that incorporate light emitting diodes in CMOS-based devices to enable random pulse generation [108]. Although there is no inherent noise source, in CMOS circuit-based [109], VLSI-based neuron [110] and semiconductor neuron [111], stochastic resonance phenomena have been confirmed by adding voltage noise from outside the systems. In semiconductor neurons, not only stochastic resonance but also output frequency locking related to coherence resonance and stochastic synchronization has been seen [111].

### 3.2. Stochastic Resonance Phenomena in Electronic Devices

It is known that stochastic resonance phenomena occur even in electronic elements and circuits that are not spike-generating excitable systems unlike many neuronal devices. For example, there are reports of stochastic resonance in Schmitt triggers that exhibit hysteresis properties and have two thresholds [112]. It has been shown that the system corresponds to the double-well potential model, which has been frequently treated as a research object of stochastic resonance, and has been found that the effect of stochastic resonance is larger than in monostable systems with only one threshold [113].

In addition, GaAs nanowire FETs have been reported to deal with stochastic resonance phenomena due to the fact that GaAs nanowire FETs are small, easily integrated, operate at room temperature, and are easy to handle. Stochastic resonances are induced in the parallel circuit (the Collins model) of the FETs using GaAs nanowire FETs. It was confirmed that signal transfer performance due to noise was gained with respect to increase in the number of parallelization [114]. Also, the phenomenon was superior to those for a parallelized linear response elements. However, pronounced was the general problem that nonlinear devices could not show better SR properties than linear devices in cases depending on the threshold of the FET and applied noise intensity. To solve this problem, Kasai et al. deliberately set different thresholds for each of the elements to be parallelized [115]. Thereby, even in the above situation, the nonlinear response system with GaAs nanowire FETs was able to maintain almost the same or higher performance than the linear response system. Until now, electronic devices used in digital equipments have been manufactured in such a way as to minimize variations in performance, but it is suggested that, on the contrary, the variation among elements may work positively in the system using stochastic resonance phenomenon.

In addition, SR phenomena were oberved in various device systems including carbon nanotube transistors [116,117,118,119] and MOSFET [113]. Furthermore, metal-insulator-transition in VO${}_{2}$ thin film device was used as a nonlinear responding system in naturally parallelized Collions model [24]. In addition, by adsorbing phosphomolybdic acid (H${}_{3}$PMo${}_{12}$O${}_{40}$) as a noise source on single-walled carbon nanotubes (SWNTs), which exhibit nonlinear response properties, a SR-like behavior was observed [120].

### 3.3. Artificial System Using Noise

There is the available publication that attempts to use stochastic resonance directly in digital signal processing systems. Generally, the presence of noise has become unavoidable as the size of computer systems is reduced and processing speeds is increased. Assuming a noisy system, it has been reported that even in such an environment, logic circuits can be operated by using stochastic resonance phenomena [121,122]. A logic circuit is one that returns an output corresponding to two inputs, but since it is also a nonlinear response system with a threshold, it can be said that this is a system in which stochastic resonance can occur. In the reported model, two digital inputs of 1 or 0 are added together to form a signal that is input to a bistable system with two thresholds and then logical operation was conducted while simultaneously applying noise of constant strength to the input signal. Although if noise intensity is too small or too large, the logic circuit will not function, it will work for applying noise with intermediate intensity. This is called logical stochastic resonance. Furthermore, it was shown that by applying noise it is possible to switch between NAND and NOR, or AND and OR logic operations depending on the input bias (or threshold).

There is an attempt to use stochastic resonance when detecting weak biological signals from the surface of the human body. By using a system of parallelized GaAs nanowire FETs, electromyography is obtained from the human upper arm via stochastic resonance phenomena with noise from living organisms [123]. It was reported that by utilizing biologically derived noise, signals with high SNR were succeeded in detection. Since in the bipolar induction method commonly used for electrocardiogram measurements, two electrodes cancel out the noise, the balance between both electrodes is important while they are vulnerable to human locomotion. For this problem, the proposed method, which uses stochastic resonance phenomena, is resistant to human movement and can more easily measure weak biological signals in the straightforward manner.

## 4. Noise in Organic Electronics

### 4.1. Organic Electronics

Organic devices have received much attention as an alternative to inorganic devices. Organic semiconductors have advantages over inorganic materials in various factors such as flexible, lightweight, stretchable, and can be deposited at low cost [124,125]. This is unlike inorganic semiconductors, which have strong covalent chemical bonds. Organic semiconductors are made up of molecules, many of which are aggregated together by weak van der Waals forces. Due to the weak bonding, it can be deposited at relatively low temperatures, and many organic materials are soluble in commonly used solvents, which lead to low cost processing strategies using spin-coating [126] and printing [127] techniques. Furthermore, the chemical properties can be changed in various ways by customizing the functional groups. The energy levels of the molecular orbitals and solubility to solvents can also be controlled [128,129]. On the other hand, since the intermolecular interaction is small, mobility of charge carriers tends to be low. The organic semiconductor film deposited using evaporation of small molecules or solvent processes for polymers contains a mixture of amorphous and/or polycrystalline materials. In many practical cases, single crystal thin films are difficult to form [130]. Furthermore, it is structurally disordered and also creates energetic disorder due to charge trap formation [131].

### 4.2. Organic Field Effect Transistor (OFET)

Organic field-effect transistors (OFETs), one of the most popular organic semiconductor devices, are switchs that can control the current value (resistance value) electrically. It has been used for applications in display backplanes, memories, actuators, physical or chemical sensors, and so on.

**Figure 4:**
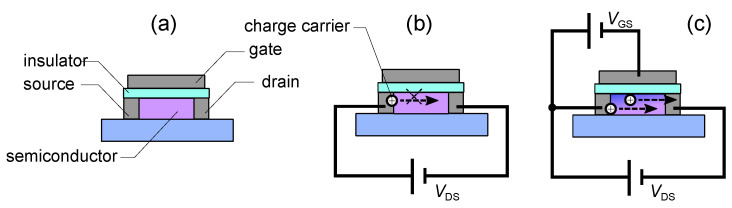
(**a**) Structure of a top-gate OFET. It has three electrodes of gate, drain, and source, and is composed of a semiconductor film and an insulating film. (**b**) No field effect by zero gate voltage. The channel is inactive at the interface between the semiconductor layer and the insulator layer, resulting in the Off-state of the OFET. (**c**) The state in which the field effect caused by the gate voltage is effective. The charge carriers transport between the drain and the source, and the OFET becomes On-state.

Typical structure of an OFET is similar to that of a MOSFET, with three terminals: source, drain, and gate electrodes. The source and drain electrodes are separated from each other by a semiconductor layer, and the source (drain) and gate electrode are separated with an insulating layer (Figure 4a). The structure in which the gate electrode is on top of the semiconductor layer and insulating layer is called the top gate structure. The structure underneath is called a bottom-gate structure. The one with the source and drain electrodes on top of the organic layer is called top contact device while The one at the bottom is called the bottom contact. Therefore, four different OFET structures can be taken depending on the position of the electrodes. The three electrodes in OFETs are generally metals. Therefore, the semiconductor-metal is in direct contact, and the higher the density of defects produced there, the more they become charge injection barriers [132] and contact resistance. In a short-channel device, this value is said to be equal to or greater than the channel resistance. Unlike OFETs, the source and drain in MOSFETs and amorphous silicon (a-Si) FETs are inorganic transistors [133], where a semiconductor that is locally doped to become a high-density charge region. In most MOSFETs, an electric voltage is applied to the gate and an inversion layer is formed in the semiconductor layer to form a channel between the source and drain and make it conductive. In the OFET, on the other hand, the gate voltage forms a storage layer in the semiconductor layer to create a channel (Figure 4c). Therefore, the channel voltage is formed by applying a negative voltage to the gate when a p-type semiconductor is used, and a positive voltage when an n-type semiconductor is used. When a channel is formed at the interface between semiconductor and insulating layers, the drain-source current $I_{\mathrm{DS}}$ increases linearly (linear region) as the drain-source voltage $V_{\mathrm{DS}}$ is applied. Using the MOSFET theory, the behavior in the linear regime is

(1) $$I_{\mathrm{DS}}=-\frac{W}{L}\mu C_{i}\left[\left(V_{\mathrm{GS}}-V_{\mathrm{th}}\right)V_{\mathrm{DS}}-\frac{V_{\mathrm{DS}}^{2}}{2}\right],$$

where *W*, *L*, $C_{i}$, $V_{\mathrm{GS}}$, $V_{\mathrm{th}}$, and μ stands for channel width, channel length, capacitance per unit area of insulating layer, gate-source voltage, threshold voltage, and mobility, respectively [134,135,136]. Here, a p-type device is assumed. Furthermore, if $V_{\mathrm{DS}}$ is applied and $V_{\mathrm{DS}}<V_{\mathrm{GS}}-V_{\mathrm{th}}$, $I_{\mathrm{DS}}$ is saturated because $I_{\mathrm{DS}}$ is no longer dependent on $V_{\mathrm{DS}}$ (saturation region) due to pinch off channel on the drain side.

(2) $$I_{\mathrm{DS}}=-\frac{W}{2L}\mu C_{i}{\left(V_{\mathrm{GS}}-V_{\mathrm{th}}\right)}^{2}$$

The threshold voltage $V_{\mathrm{th}}$ is the value of $V_{\mathrm{GS}}$ such that the semiconductor layer begins to form a channel and becomes a conducting state. If $V_{\mathrm{GS}}$ is below the threshold ($V_{\mathrm{GS}}>V_{\mathrm{th}}$ for the p-type FET), then ideally

(3) $$I_{\mathrm{DS}}=0.$$

**Figure 5:**
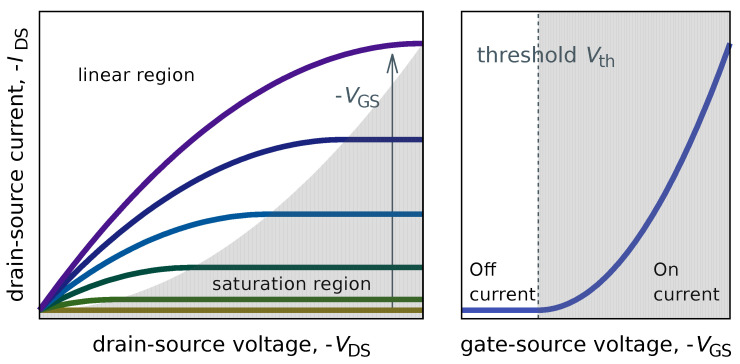
(**left**) Schematical illustration of the output characteristics of a p-type OFET (drain-source current $I_{\mathrm{DS}}$ vs. drain-source voltage $V_{\mathrm{DS}}$). As $V_{\mathrm{DS}}$ is increased, the system enters the saturation region from the linear region. Also, as $V_{\mathrm{GS}}$ is applied, $I_{\mathrm{DS}}$ increases due to the electric field effect. (**right**) transfer characteristic (drain-source current $I_{\mathrm{DS}}$ vs. gate-drain voltage $V_{\mathrm{GS}}$). $-I_{\mathrm{DS}}$ is plotted as a function of $-V_{\mathrm{GS}}$ under the condition of a constant $V_{\mathrm{DS}}$. Typical nonlinear response with a threshold can be seen. The electric current when it is less than the threshold is called Off-current, and the current when it is more than the threshold is called On-current.

If we draw the transfer characteristic (the graph of $I_{\mathrm{DS}}$ vs. $V_{\mathrm{GS}}$) under the constant $V_{\mathrm{DS}}$, for p-type FETs, nonlinear characteristics with a threshold can be obtained (Figure 5 (right)), where electric current of $I_{\mathrm{DS}}$ for the condition of $V_{\mathrm{GS}}\leq V_{\mathrm{th}}$ (On-state) whilest $I_{\mathrm{DS}}$ does not flow for the condition of $V_{\mathrm{GS}}>V_{\mathrm{th}}$.

The electrical characteristics of qualitative OFETs, where the output and transfer characteristics of the p-type OFET can be plotted as shown in Figure 5, can be reproduced using Equations (1) or (2) or (3). However, although the models presented in Equations (1)–(3) can roughly represent the properties of OFETs, they often do not precisely reproduce them, and various models have been proposed [137,138,139,140,141].

One of challenges to the practical application of OFETs is mobility [142]. A poly thiophene-based OFET fabricated in 1986 had a mobility of $10^{-5}$ cm${}^{2}$/Vs [143]. More recently, OFETs with mobility of more than 1 cm${}^{2}$/Vs have been reported [144], and mobility equal to or higher than that of a-Si FETs (Si TFT: 0.5–1 cm${}^{2}$/Vs) is being achieved. However, generally, compared to inorganic FETs, OFETs have lower mobility and smaller On-Off ratio. In other words, this drawback would cause low sensitivity and responsiveness to sensory signals.

### 4.3. Poly(hexylthiophene)

Poly(3-hexylthiophene) [P3HT], one of poly(alkylthiophene)s, has often been used as a polymer semiconductor. Regioregular version of this polymer semicondutor, abbr. RR-P3HT, shows relatively high hole mobility due to high planarity maintained by low steric hindrance of thiophene rings, π-conjugated system extended by intermolecular π-π stacking, and high crystallinity by its self-assembling characteristic [145].

**Figure 6:**
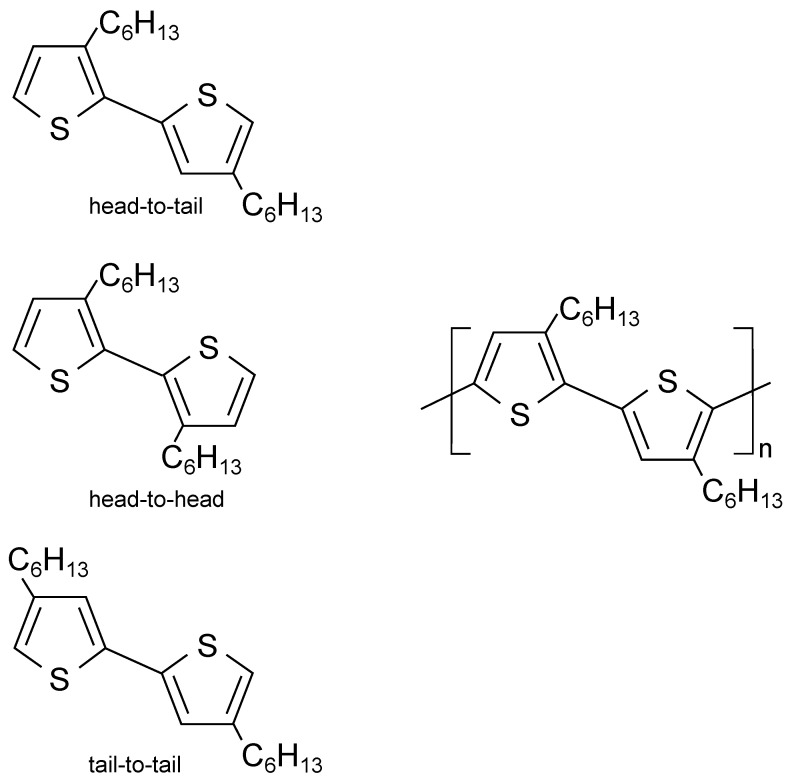
Chemical structure of 3-hexylthiophene dimers in head-to-tail (**top left**), head-to-head (**middle left**), tail-to-tail (**bottom left**) bonds, and regioregular poly(3-hexylthiophene-2,5-diyl)[RR-P3HT] (**right**).

Since RR-P3HT molecules do not have a donor-acceptor moiety, it has a larger band gap compared to donor-acceptor polymer semiconductors [146]. However, P3HT has a lower glass transition temperature $T_{g}$ than donor-acceptor polymers, which provides RR-P3HT as a material of relatively non-brittle material and of a high tensile modulus [147]. As a result, cracks are less likely to occur during the fabrication of electronic devices or during use, and the adhesive surfaces with the other materials are less likely to peel off. P3HT affects the formation of ordered domains when regioregularity is high [148] and increases mobility [145] (see Figure 6 (top left) for high regioregularity, i.e. head-to-tail coupling of monomer units). The delocalized molecular orbitals become more overlapped between neighboring polymer chains, which promotes charge transport. Mobilities of 0.05–0.1 cm${}^{2}$/Vs have been observed in OFETs with RR-P3HT (Figure 6 (right)) [145].

Furthermore, 1.2 cm${}^{2}$/Vs was found in OFETs with defect-free regio-regularity 100% P3HT [149]. On the other hand, in regio-random P3HT [RRa-P3HT] with low regioregularity, where head-to-tail and heat-to-head (tail-to-tail) bonds are irregularly present. The disorder due to the twisting of the thiophene ring increases, resulting in irregular packing and The mobility becomes lower ($10^{-5}–10^{-4}$ cm${}^{2}$/Vs) [150].

There is anisotropy in the conductivity of the charge carriers in P3HT [145]. The mobility along the main chain in RR-P3HT is high and the crystallites show the highest mobility. The mobility in the π-π stacking direction is higher than that in the direction parallel to the side chains of P3HT. Therefore, the degree of crystallinity and the orientation of crystal grains can be controlled. This is important for the control of electrical characteristics. The degree of crystallinity and crystal orientation of P3HT is affected by regioregularity, molecular weight, hydrophilicity of the substrate surface, and the casting solvent [151]. When the molecular weight is large, the collapsed face-on structure is more dominant than the standing edge-on structure of the thiophene ring on the substrate. The bulk tends to be face-on rich and the thin film tends to be edge-on rich, and the high volatility of the casting solvent tends to cause the face-on structure. The edge-on structure is considered to be thermodynamically stable, while the face-on structure is considered to be kinetically stable.

### 4.4. Noise in OFET

In semiconductor devices, electrical noise is classified [152]. The noises that have been identified in organic semiconductor devices include thermal noise, shot noise, random telegraph noise, 1/f-noise [153], where *f* is frequency. One of them that has been identified in many OFETs is 1/f noise [154]. The 1/f noise is the noise whose spectrum is 1/f. It is sometimes referred to as low-frequency noise, but the origin of the generation is not well understood. Hooge’s empirical relationships identified in inorganic transistors including MOSFETs [155,156] is also used for noise analysis in OFETs. Hooge’s empirical relation is

(4) $$\frac{S_{R}}{R^{2}}=\frac{S_{I}}{I^{2}}=\frac{S_{V}}{V^{2}}=\frac{\alpha_{H}}{fN_{c}}.$$

This is indicated by $S_{R}/R^{2},S_{I}/I^{2}$, and $S_{V}/V^{2}$ are the relative noise intensities of the resistance, current, and voltage, respectively, $\alpha_{H}$ is the Hooge parameter, and $N_{c}$ is the total number of free charge carriers in the material. Although the constant $\alpha_{H}$ is known to depend on temperature and magnetic field as well as crystallinity and material type. The larger $\alpha_{H}$ gives the larger noise intensity. If the more heterogeneous the structure of the system is, the larger the value of $\alpha_{H}$ tends to be. The $\alpha_{H}$ in a homogeneous and ohmic sample is approximately $2\times 10^{-3}$ or within two orders of magnitude of that, but for strong heterogeneity and disorder, it is much larger than $2\times 10^{-1}$. Organic semiconductor devices often fall into the latter category and tend to have higher 1/f noise compared to inorganic devices [154].

The origin of the 1/f noise in OFET is thought to be mobility or carrier number fluctuations, and the formula derived from Hooge’s empirical relation

(5) $$\frac{S_{I}}{I^{2}}\propto \frac{1}{N_{c}}=\frac{q\mu \rho }{V}$$

is often used, where μ, ρ, and *V* are free carrier mobility, resistivity, and volume of the resistive element, respectively. In a study dealing with pentacene transistors, 1/f noise of $\alpha_{H}=0.01–0.08$ was observed [157], and mobility fluctuations were considered to be the predominant [158]. In poly(3-hexadecylthiophene), it has been suggested that the low-frequency noise in the transistor is derived from mobility fluctuations due to hopping conduction process [159].

On the contrary, the carrier trapping and detrapping at the grain boundary can lead to carrier number fluctuations are said to occur [160]. The γ of $1/f^{\gamma }$ noise has been observed to increase from the linear to the saturation region of OFETs [161,162], while this has been explained to be due to the entrapment of deeper traps at grain boundaries [162]. Since OFETs driven in the saturation region do not form the storage layer in the semiconductor near the drain electrode due to pinch off, the Fermi level is located near the center of the HOMO-LUMO gap and deep traps near the HOMO energetically approaches the Fermi level. This allows the carriers to occupy the trap level and the trap-detrapping process is thermally accelerated. This trap-detrapping process works for noise generation. Since the trap-detrapping process in the deep traps is slow, which increases the value of γ.

There is also a report on the 1/f noise property with respect to mobility and film thickness of OFETs. By changing the number of stacked semiconductor layers, the thickness of the active layer can be varied, and the effect of varying the mobility on the noise intensity has been studied [163]. As a result, when the film thickness is less than 20 nm, the relationship between noise intensity and mobility is $S_{I}/I^{2}\propto \mu^{-\omega }$. It was found that the percolation model with a mixture of conductive and insulating components results in the relation $S_{I}/I^{2}\propto \rho^{\omega }$. Indeed, assuming that the charge carrier density is constant, the experimentally confirmed relationship followed a percolation model. Therefore, the presence of resistive barriers due to randomly distributed grain boundaries infers that it contributes to the hopping conduction and effectiveness for noise generation.

**Figure 7:**
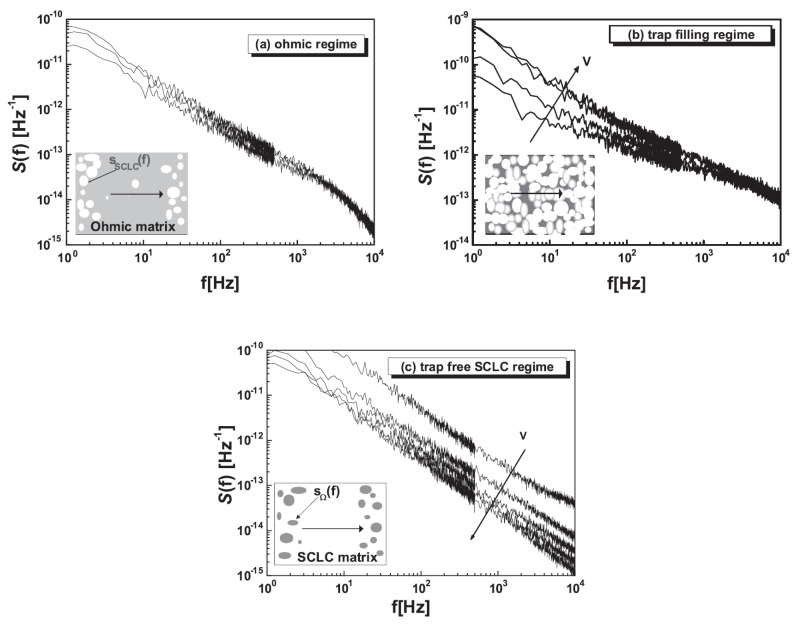
Power spectral density S(f) of relative current noise in Au/tetracene/Al devices. (**a**) Ohmic regime: S(f) does not change with voltage *V*. (**b**) Trap−filling regime: during the trap−filling transition, the trap S(f) increases rapidly with *V*. (**c**) Space−charge limited current regime: S(f) decreases approximately according to 1/V. (inset) The horizontal arrow indicates the direction of the current. The white region indicates trapped carriers, which are insulating regions characterized by $s_{\mathrm{SCLC}}\left(f\right)$ noise. Dark regions indicate vacant traps and are conductive regions characterized by $s_{\Omega }\left(f\right)$ noise: [164] adapted with permission from *Physical Review Letters* **2005**, *95*, 236601. Copyright 2005 American Physical Society.

Furthermore, percolation effects have been shown to be present in the voltage-dependent trap filling mechanism [164]. Space charge limited current (SCLC) behavior is known to occur in a vertical-type diode with Au/pentacene/Al structure [165], and the current characteristics are varied with respect to applied voltages, which are divided into regimes called ohmic, trap filling, and SCLC regions. It has been confirmed that the current noise in each region shows different voltage dependence. The current noise intensity in the ohmic region is independent of the voltage (Figure 7a), proportional to applied voltage in the trap filling region (Figure 7b) and inversely proportional to applied voltage in the SCLC region (Figure 7c). In other words, the noise intensity is maximum in the trap filling region. This behavior can be explained using a percolation model. In the ohmic region, where drift conduction due to the applied electric field occurs, the semiconductor is treated as a conducting state (Figure 7a), and in the SCLC region, the deep traps are all filled by injected carriers, which can be considered as insulating state (Figure 7c). Trap filling regime, which is located in the middle of ohmic and SCLC regimes, is considered to be in the situation that the semiconductor has a percolation structure with conductive and insulating components, and it can be explained that a large noise is generated by this structure (Figure 7b).

Also, one of polyalkylthiophenes, RR-poly(3-decylthiophene) [RR-P3DT] has been received the focus of attention as a possible material for intentional noise generation [25]. The current characteristics of the vertical-type devices with Au/RR-P3DT/Au structure were investigated, and larger current fluctuation was observed than that of Au/RR-P3HT/Au devices. In particular, when the applied voltage is the voltage at which the trap filling transition occurs ($V_{\mathrm{TFT}}$), it is found to output a large current noise. The $V_{\mathrm{TFT}}$ decreased with increasing temperature. This phenomenon may be derived from thermally activated stochastic transition of charge carriers between several conduction paths which is also drifted by applied electric field. In addition, it has been suggested that the carrier transport process in RR-P3DT involves the twisting motion of the main chain by the thiophene ring of P3DT and the molecular motion of the alkyl side chains, which is related to order-disorder phase transition (ODT) associated with the twisting motion of the main chain. It is believed that noise is generated by the formation of shallow traps due to the ODT and thermally activated and electric field accerelated detrapping of charge carriers.

**Figure 8:**
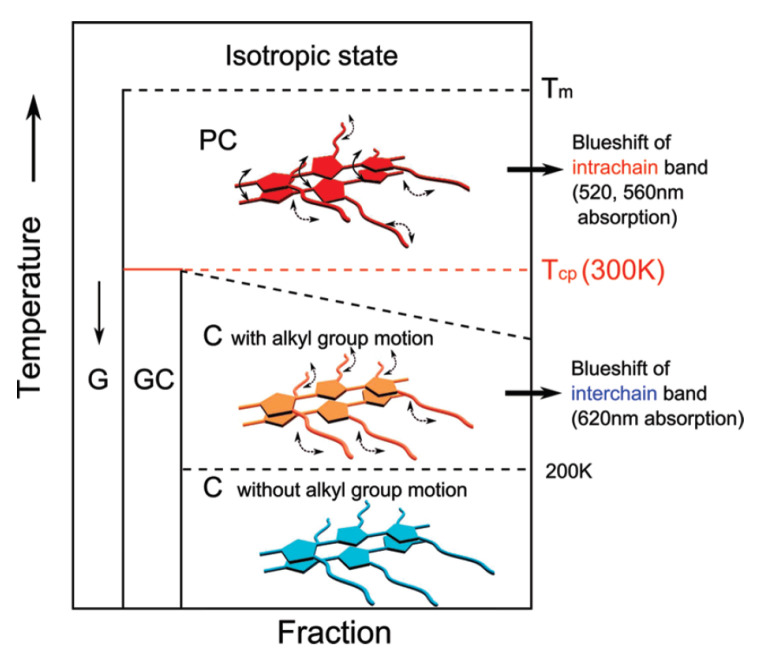
State diagram of RR-P3HT. The width of horizontal axis shows the qualitative fraction of thermodynamic phase or glassy states included: [168] adapted with permission from *The Journal of Physical Chemistry B* **2010**, *114*, 1241. Copyright 2010 American Chemical Society.

In RR-P3HT, the phase transition from the crystalline (C) phase to the plastic crystalline (PC) phase continuously occurs between 240 and 300 K, where the torsional motion of the thiophene rings is intiated [166,167,168]. In this case, C and PC phases coexist until *ca.* 300 K (Figure 8). Similarly, ODT occurs in P3DT from 303 to 353 K. However, the structural change due to ODT is more reflected in the electrical properties than in P3HT. This dynamic disorder can also be used as a potential source of noise generation material.

## 5. Bio-Mimicking Noise-Driven Signal Processing

By mimicking the information processing mechanisms of living organisms, it may be possible to construct systems with functions and performance that have been difficult to achieve with digital processing systems. In particular, we focus on the use of noise, and deal with stochastic resonance phenomena and consider their application to electronic systems. In many cases, noise has been perceived as unnecessary, degrading the system, and consideration has been given to how to eliminate noise. However, noise can be present in any situation, and if we can tolerate it and make it work in a positive way this can lead to the construction of an artificial system with low energy consumption.

### 5.1. Stochastic Resonance Phenomena Due to Internal Noise

As we have discussed, a number of theoretical studies have shown that in a system modeled with nonlinear response properties, such as neurons, various characteristics and functions of stochastic resonance phenomena are presented and investigated with related brain function. Since it is difficult to confirm the stochastic resonance phenomenon with internal noise, it is not clear whether stochastic resonance contributes to the information processing in the brain whilst physiological experiments with practical organisms and cells have been conducted.

There are also approaches to create artificial neurons by electronic systems and investigate their relation to stochastic resonance phenomena, but most of experiments are performed with external noise. There have been reports on systems with internal noise, such as the two-terminal device [120], which has a molecule as a noise source, but the scalability of that system is low when a network formation with an ensemble of the device elements is considered. Most of reports in practical electronic devices such as transistors involves stochastic resonances due to the application of external noise. As described above, there is no research that deals with stochastic resonance phenomena experimentally in systems with internal noise, especially their networked systems.

### 5.2. Characteristics of the Desired System

In order to understand the information processing mechanism using the stochastic resonance phenomenon, and to create biomimetic information processing systems, devices with the following characteristics are considered to be effective.

(a) system with internal noise
(b) nonlinear response characteristics with respect to external stimuli
(c) one-directional signal transmission

(a) If the device has internal noise, this noise may be able to emerge stochastic resonance phenomena and thus to improve the signal transmission performance. In particular, when reproducing the Collins model, where the effect of stochastic resonance is enhanced. Since each device element to be parallelized needs to have independent noise, it could be useful for the device to have its own inherent noise. In addition, if we can form a network with multiple device elements and can investigate the relationship between the function of the network and the stochastic resonance phenomenon caused by the internal noise, this may provide insight into the contribution of stochastic resonance to brain function. (b) Nonlinear response properties with respect to external stimuli are known to be a necessary condition to induce stochastic resonance phenomena. In a linear system added with external noise, the noise will appear on the output as it is, it will only induce a degradation in the system’s performance. If the system has a potential barrier, such as a threshold unit, noise can positively be used to overcome that barrier, and stochastic resonance is emerged. (c) One-directional transmission of signals can be achieved by separating the input signal from the output signal and preventing the signal from flowing backwards, which is also one of characteristics of a synapse. With this mechanism, we can create a complex network of feedback circuits as well as feed-forward ones, which creates complex spatio-temporal patterns compared to two-directional signal transmitting systems that simply spread the signal in a diffusive manner.

Among electronic devices, transistors are those that (b) exhibit nonlinear response characteristic and (c) transmit signals in one direction. Among transistors, organic field-effect transistors (OFETs), which are made from organic semiconductors, have the potential to (a) impart large internal noise.

When the gate voltage ($V_{\mathrm{GS}}$) over a threshold voltage $V_{\mathrm{th}}$ is applied to the OFET as an input signal, the drain-source current ($I_{\mathrm{DS}}$) increases, indicating that OFET exhibits a nonlinear response characteristic with threshold (Figure 5 (right)). When subthreshold signal is input, the system is insensitive to input signal. But It is expected that the addition of noise to the subthreshold input signal will cause total input signal to exceed the threshold and then responsiveness of the system to increase. In other words, OFET is a system in which stochastic resonance can be expected to occur.

Since the presence of the insulating film in the FET makes the input impedance to $V_{\mathrm{GS}}$ very high and also since the voltage to current conversion is performed by the field effect, the reverse flow of the output signal is prevented and the mechanism of one-directional signal transmission is achieved. Also, compared to inorganic materials, organic semiconductors are expected to have greater noise in OFETs due to many factors including static and dynamic disorder.

### 5.3. Stochastic Resonance in OFETs

As we have discussed, OFETs are expected to have relatively large internal noise. Therefore, if we can clarify the effect of the internal noise on the stochastic resonance, it leads to discovering the function and availability of internal noise of the device system. Furthermore, since OFETs, which can transmit signals in one-direction, are thought to be relatively easy to construct a network system, it may help to elucidate the role of noise in more complex systems such as neural networks.

However, in general, OFETs are likely to have lower mobility than inorganic transistors such as MOSFETs. This means that the electric field responses of OFETs tend to be low due to its weak nonlinearity. Hence, it is unclear whether stochastic resonance is manifested in OFET such as inorganic FETs. Therefore, the purpose of this review is to investigate whether stochastic resonance phenomenon can be confirmed by applying external noise to the OFET. Investigation of the effect of noise in the physical system, such as internal noise in OFETs, is also reviewed. Furthermore, the effect of internal noise on the stochastic resonance is numerically analyzed.

## 6. Observation of Stochastic Resonance in Organic Field-Effect Transistors

Here, we describe an experimental realization of stochastic resonance by externally applying an electric field noise to typical OFET using RR-P3HT. Since the frequency band that the RR-P3HT-based OFET system can drive is narrow, load resistance in the drain ground circuit has been investigated, aiming to widen the band further. OFETs with organic semiconductors are also expected to have large noise [154], and it is clarified whether the noise may act on the stochastic resonance phenomenon and characterized the stochastic resonance behavior observed in OFETs.

In addition, the manifestation of stochastic resonance phenomena requires the system to exhibit a threshold-like nonlinear response [40]. In order to confirm whether the stochastic resonance phenomenon is observed in OFETs that show different transfer characteristics, the conditions under which stochastic resonance is effective in OFET have been explored [28].

### 6.1. Measurement Method

**Figure 9:**
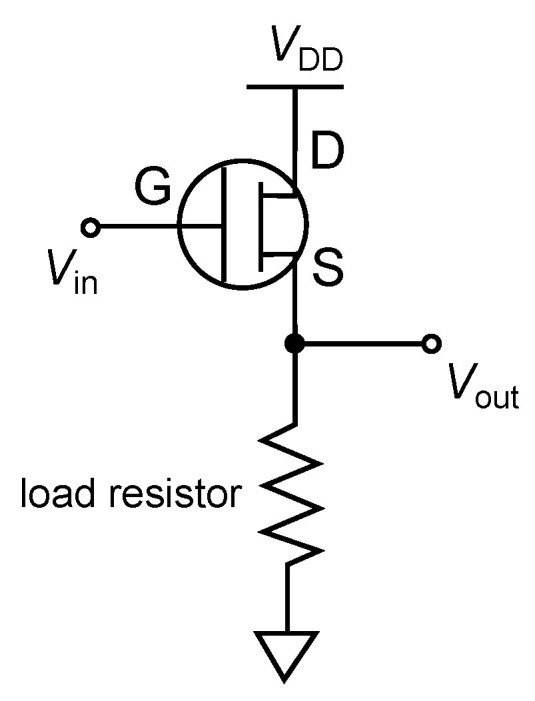
Drain-grounded (source follower) circuit.

In order to confirm whether the stochastic resonance phenomenon occurs by applying external noise to the RR-P3HT based OFET, the periodic input signal and the noise voltage were input to the drain ground circuit (Figure 9), which was composed of the OFET and the load resistor.

**Figure 10:**
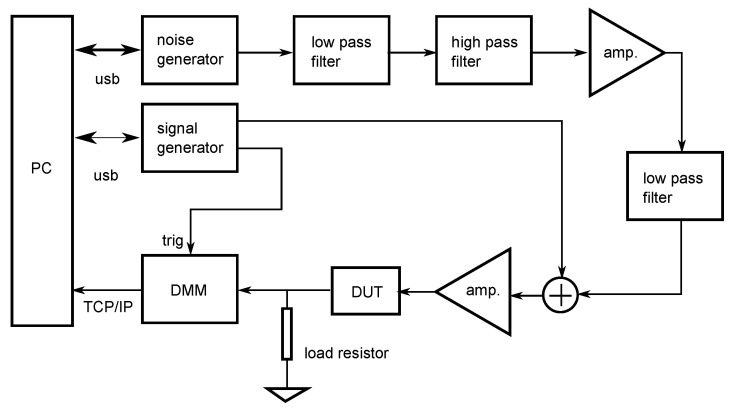
Block diagram of the apparatus used in stochastic resonance experiments to measure input-output mutual correlation. DUT (device under test) is a RR-P3HT-based OFET, of which a drain-grounded circuit is composed together with a load resistor. The frequency band for noise was filtered and the filtered noise was added to the input signal and amplified to be input to the gate electrode of the OFET [169] adapted with permission from doctoral dissertation of Yoshiharu Suzuki. Copyright 2019 Gunma University.

Gaussian white noise was used for the experiments and then, the output signal of the OFET circuit was measured. The configuration of the equipment used for the experiment of the stochastic resonance phenomenon is shown in Figure 10.

The periodic input signal and Gaussian white noise applied to the OFET system were generated using an arbitrary waveform generator (3390, Keithley for the input signal and 33210A, Keysight Tech. for the noise), added, and amplified by a high-speed bipolar amplifier (HS4011, NF). A sine wave and a square wave of the frequeny of 0.5 Hz and the duty ratio of 80 % of the square wave were used as input periodic signals. The bandwidth of the noise was set to 0.05–48 Hz. The noise generated by the arbitrary waveform generator was upper bounded in frequency by an RC low-pass filter. A DC power supply (PSF-400H, TEXIO) was used to add −15 V to the $V_{\mathrm{DD}}$ power supply to the circuit. The measurement of the output signal of the OFET circuit was performed with a digital multimeter (33410A, Keysight Tech.) and the sampling frequency was 50 Hz. As a measure of the signal transmission performance of the OFET system, the correlation value (correlation coefficient) ρ between the input and output signals was used.

(6) $$\rho =\frac{\frac{1}{t_{M}}\int_{0}^{t_{M}}\left[V_{\mathrm{out}}\left(t\right)-\bar{V_{\mathrm{out}}\left(t\right)}\right][R\left(t\right)-\bar{R(t)}]dt}{\sqrt{\frac{1}{t_{M}}\int_{0}^{t_{M}}\left[V_{\mathrm{out}}\left(t\right)-\bar{V_{\mathrm{out}}\left(t\right)}\right]{}^{2}dt}\sqrt{\frac{1}{t_{M}}\int_{0}^{t_{M}}\left[R\left(t\right)-\bar{R(t)}\right]{}^{2}dt}},$$

where $t_{M}$ is the measurement time, R(t) is the input (reference) signal, $V_{\mathrm{out}}\left(t\right)$ is the output signal, and $\bar{R(t)},\bar{V_{\mathrm{out}}\left(t\right)}$ are the average values of the input and output signals, respectively.

**Figure 11:**
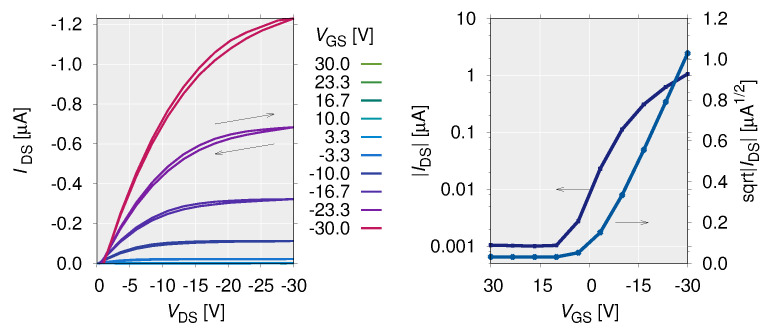
Output characteristic $I_{\mathrm{DS}}$ vs. $V_{\mathrm{DS}}$ (**left**) and transfer characteristic $I_{\mathrm{DS}}$ vs. $V_{\mathrm{GS}}$ at $V_{\mathrm{DS}}$ = −15.6 V (**right**) for the RR−P3HT−based OFET used in stochastic resonance experiments [26]. Adapted with permission from *Applied Physics Letters* **2016**, *109*, 093702. Copyright 2016 American Institute of Physics.

The electrical characteristics of the OFET are shown in Figure 11. With using Equation (2), the field-effect mobility and threshold voltage are calculated as $\mu =3\times 10^{-3}$ cm${}^{2}$/Vs and $V_{\mathrm{th}}=-0.45$ V, respectively.

### 6.2. Load Resistance Study

The summed signal of a sinusoidal wave of 6 V amplitude with external noise, was input to the drain-grounded circuit composed of the RR-P3HT-based OFET and load resistance of 4.4 kΩ. The signal transfer performance of the OFET system was improved by the applied external noise and therefore the stochastic resonance phenomenon was observed in the OFET. On the other hand, when the load resistance is 47 kΩ, the correlation value decreases due to the application of external noise. The correlation value is brought close to zero with respect to external noise intensity and this trend was unchanged with varying the DC bias and input signal amplitude. Since the channel resistance of the OFET is relatively large, a drain-grounded circuit using a larger load resistance will result in a larger output than one with a smaller load resistance. At the same time, however, a high pass filter by the RC circuit which was formed by the gate capacitor in the OFET and the load resistor has deteriorated the frequency characteristics of the drain-grounded circuit, which results in the conjecture that stochastic resonance phenomenon was not seen when the load resistance is increased. Inputs with a frequency higher than the cutoff frequency of the RC circuit pass through the circuit and appear at the output. This means that regardless of the nonlinear characteristics of the OFET, the high-frequency input is transferred linearly. In these measurements, the driving frequency of the OFET circuit was widened when the value of the load resistance used was small (4.4 kΩ), and the stochastic resonance phenomenon was observed. However, since further reduction of the load resistance would give attenuation of output intensity of the OFET system, further improvement in system responsiveness by adjusting the load resistance is unlikely.

Although the obtained results from the stochastic resonance experiments are consistent with previous experiments [24,113,114,115,116,117,118,119], the bell-shaped curve with a clear peak in input-output correlation value was not confirmed. The stochastic resonance was also observed even when a square wave was used as the input signal, and the similar behavior as for sine waves was observed when using a square wave.

### 6.3. Stochastic Resonance Phenomena in OFET Systems

#### 6.3.1. The Behavior of Stochastic Resonance Phenomena

**Figure 12:**
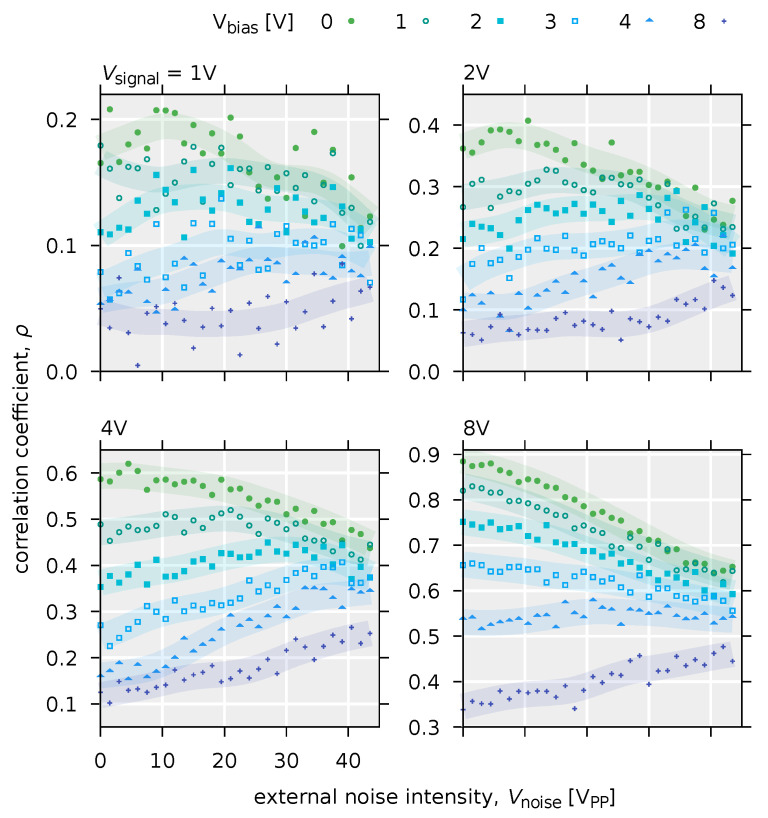
Input-output correlation coefficients when a square wave is input to a drain-grounded circuit with a RR-P3HT-based OFET and a 4.4 kΩ load resistor, and the external noise intensity applied simultaneously is increased. In each condition of square wave amplitude $V_{\mathrm{signal}}=$ 1, 2, 4, and 8 V. The results are shown when the DC bias $V_{\mathrm{bias}}=$ 0, 1, 2, 3, 4, and 8 V. The points are the averaged values of the three trial measurements, and the curve is the guides for eyes [26] adapted with permission from *Applied Physics Letters* **2016**, *109*, 093702. Copyright 2016 American Institute of Physics.

**Figure 13:**
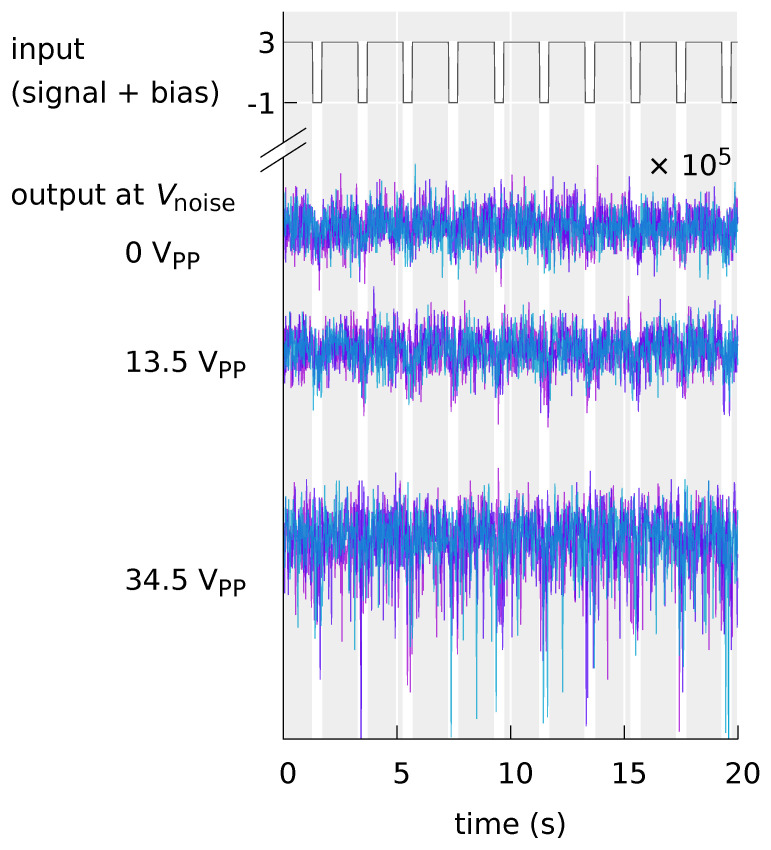
Output signal waveforms of the RR-P3HT-based OFET system when a bell-shaped curve of correlation values is observed at Figure 12 ($V_{\mathrm{signal}}=2$ V, $V_{\mathrm{bias}}=1$ V). The output signals of the three trials are superimposed on each other. When the external noise is optimal noise intensity $V_{\mathrm{noise}}$ of 13.5 $V_{\mathrm{PP}}$, the output waveform more similar to the input signal than those for the other noise intensities is confirmed [169]. Adapted with permission from doctoral dissertation of Yoshiharu Suzuki. Copyright 2019 Gunma University.

Figure 12 shows the input-output correlations with respect to external noise intensity. When $V_{\mathrm{signal}}=2$ V or when $V_{\mathrm{signal}}=$ 8 V and $V_{\mathrm{bias}}=$ 4 or 8 V, the correlation value ρ increases with increase in external noise intensity $V_{\mathrm{noise}}$. In particular, a balanced bell-shaped curve of the input-output correlation values is observed when $V_{\mathrm{signal}}=$ 2 V and $V_{\mathrm{bias}}\leq$ 2 V. This is typical evidence of the manifestation of stochastic resonance phenomena (Figure 13) [40].

#### 6.3.2. Modeling the OFET System

In order to gain further insight into the behavior of stochastic resonances in OFET systems, the numerical simulation of the input-output correlation value ρ as a function of the external noise intensity $V_{\mathrm{noise}}$ is performed. In preparation, the input-output characteristics of the OFET system were modeled. The transfer characteristics of OFET are

(7) $$I_{\mathrm{DS}}=\left\{\begin{array}{cc} -\frac{W}{2L}\mu C_{i}{\left(V_{\mathrm{GS}}-V_{\mathrm{th}}\right)}^{2}\; & \left(V_{\mathrm{DS}}\left(t\right)<V_{\mathrm{th}}\right)\\ 0\; & \left(\mathrm{otherwise}\right) \end{array}\right.$$

**Figure 14:**
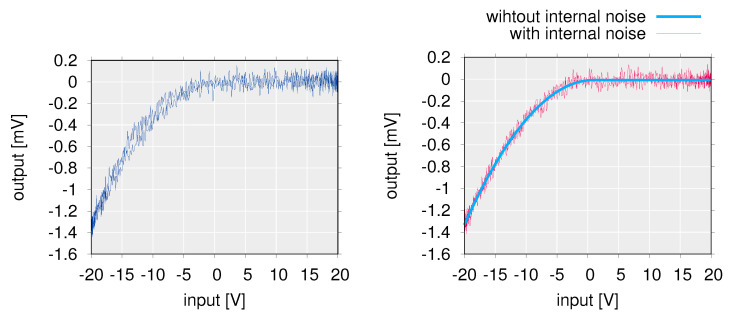
(**left**) Input-output characteristics of a drain ground circuit with a RR-P3HT-based OFET and a load resistance of 4.4 kΩ. (**right**) Input and output characteristics of OFET systems without and with internal noise, modeled by Equations (8) and (9) [26]. Adapted with permission from *Applied Physics Letters* **2016**, *109*, 093702. Copyright 2016 American Institute of Physics.

Since it can be modeled by $I_{\mathrm{DS}}$: drain-source current, *W*: channel width, *L*: channel length, $C_{i}$: capacitance per unit area of dielectric layer, $V_{\mathrm{GS}}$: gate-source voltage, and $V_{\mathrm{th}}$: threshold voltage), the output $V_{\mathrm{out}}\left(t\right)$ of the OFET system is expressed by the following equation.

(8) $$V_{\mathrm{out}}\left(t\right)=\left\{\begin{array}{cc} -A{\left(V_{\mathrm{in}}\left(t\right)-V_{\mathrm{th}}\right)}^{2}+B & \left(V_{\mathrm{in}}\left(t\right)<V_{\mathrm{th}}\right)\\ B & (\mathrm{otherwise}), \end{array}\right.$$

where $V_{\mathrm{in}}\left(t\right)=R\left(t\right)+\xi_{\mathrm{ext}}\left(t\right)$ is the input voltage and $\xi_{\mathrm{ext}}$ denotes external noise and satisfies $\langle \xi_{\mathrm{ext}}\left(t\right)\xi_{\mathrm{ext}}\left(t^{\prime }\right)\rangle =\sigma_{\mathrm{ext}}^{2}\delta \left(t-t^{\prime }\right)$. Note that $\sigma_{\mathrm{ext}}$ is the root mean square (RMS) intensity of external noise and δ(t) is Dirac’s delta function. Also, *A* and *B* are fitting parameters that are constants. From the experimentally measured input-output characteristics of the OFET system (Figure 14 (left)) $A=3.0\times 10^{-6}$ V${}^{-1}$, $B=-7.6\times 10^{-6}$ V, and $V_{\mathrm{th}}=9.4\times 10^{-1}$ V.

Furthermore, in order to better simulate the input and output characteristics of the real RR-P3HT-based OFET system, Suzuki et al. have also defined a stochastic model shown by the following equation as well as the deterministic model by Equation (8) [26]. The stochastic model consists of a random variable of constant strength added to Equation (8) as internal noise.

(9) $$V_{\mathrm{out}}\left(t\right)=\left\{\begin{array}{cc} -A{\left(V_{\mathrm{in}}\left(t\right)-V_{\mathrm{th}}\right)}^{2}+B+\xi_{\mathrm{int}}\left(t\right) & \left(V_{\mathrm{in}}\left(t\right)<V_{\mathrm{th}}\right)\\ B+\xi_{\mathrm{int}}\left(t\right) & (\mathrm{otherwise}). \end{array}\right.$$

$\xi_{\mathrm{int}}\left(t\right)$ is zero-mean Gaussian white noise, satisfying $\langle \xi_{\mathrm{int}}\left(t\right)\xi_{\mathrm{int}}\left(t^{\prime }\right)\rangle =\sigma_{\mathrm{int}}^{2}\delta \left(t-t^{\prime }\right)$. $\sigma_{\mathrm{int}}$ is the internal noise RMS intensity, and set to $5.0\times 10^{-5}$ V. The distribution functions of the external and internal noise are $1/\sqrt{2\pi \sigma_{\mathrm{ext}}^{2}}$$\exp(-\xi_{\mathrm{ext}}^{2}/\left(2\sigma_{\mathrm{ext}}^{2}\right))$ and $1/\sqrt{2\pi \sigma_{\mathrm{int}}^{2}}$$\exp(-\xi_{\mathrm{int}}^{2}/\left(2\sigma_{\mathrm{int}}^{2}\right))$, respectively. Figure 14 (right) was obtained by plotting the input-output characteristics of the OFET system modeled by Equations (8) and (9), respectively.

#### 6.3.3. Numerical Simulation of Stochastic Resonance and Structure of OFET System

**Figure 15:**
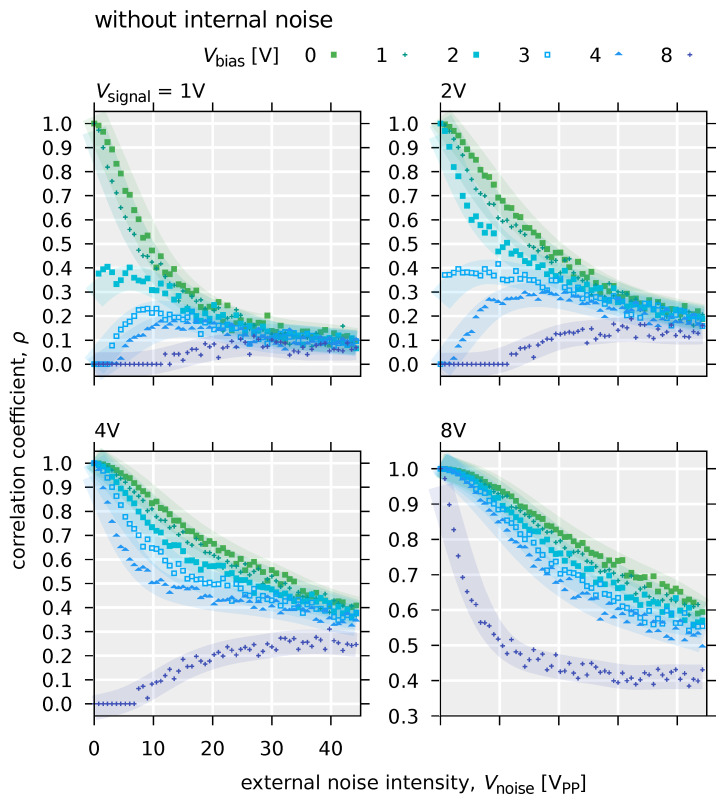
Input-output correlation coefficients when a square wave is input to a modeled OFET system with no internal noise (Equation (8)) and the external noise intensity applied simultaneously is increased. In each condition of square wave amplitude $V_{\mathrm{signal}}=$ 1, 2, 4, and 8 V, the results are shown when the DC bias $V_{\mathrm{bias}}=$ 0, 1, 2, 3, 4, and 8 V. The points are the average of the results of three numerical calculations, and the curve is the guides for eyes [26]. Adapted with permission from *Applied Physics Letters* **2016**, *109*, 093702. Copyright 2016 American Institute of Physics.

**Figure 16:**
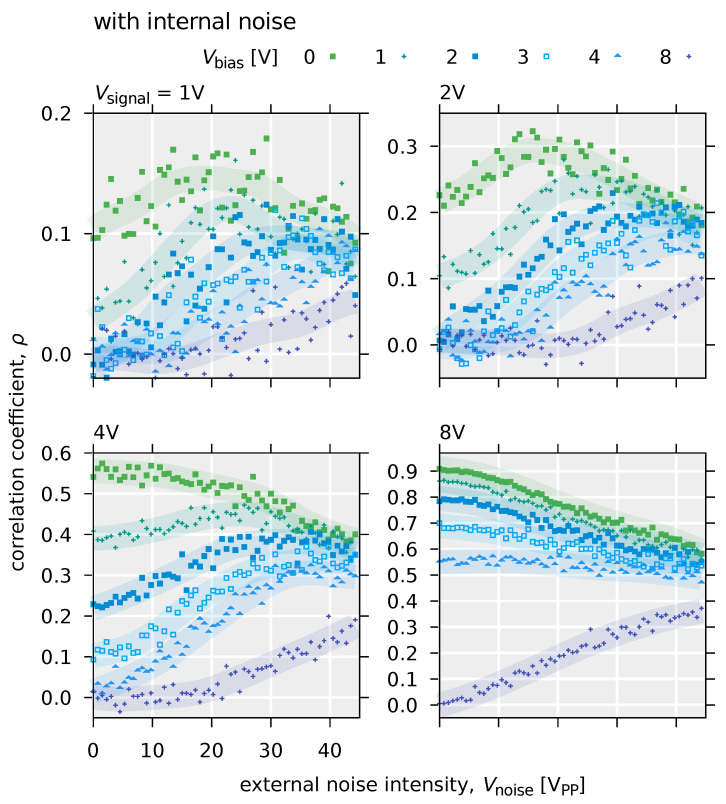
Input-output correlation coefficients for an OFET system with modeled internal noise (Equation (9)) when a square wave is input and the external noise intensity applied simultaneously is increased. In each condition of square wave amplitude $V_{\mathrm{signal}}=$ 1, 2, 4, and 8 V, the results are shown when the DC bias $V_{\mathrm{bias}}=$ 0, 1, 2, 3, 4, and 8 V. The points are the average of the results of three numerical calculations, and the curve is the guides for eyes [26]. Adapted with permission from *Applied Physics Letters* **2016**, *109*, 093702. Copyright 2016 American Institute of Physics.

Figure 15 and Figure 16 show the results of the simulation of the stochastic resonance using the input-output characteristics of the OFET system defined by Equations (8) and (9), respectively.

**Figure 17:**
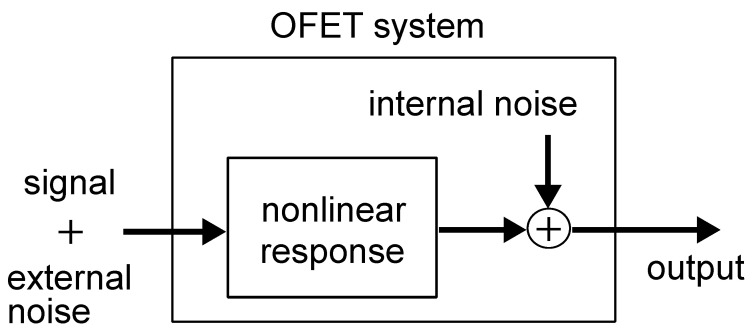
Structural diagram of the RR-P3HT-based OFET system when a signal and external noise are applied. The input is processed by the nonlinear response characteristics of the OFET, and then the internal noise is added and output [169]. Adapted with permission from doctoral dissertation of Yoshiharu Suzuki. Copyright 2019 Gunma University.

In the case of a deterministic model without internal noise (Equation (8)), for small input signals, the increase in the correlation value between the input and output signals due to external noise is clearly visible in the small range of the external noise intensity. For example, this can be pronounced when $V_{\mathrm{signal}}=2$ V and $V_{\mathrm{bias}}=$ 3, 4, and 8 V. Furthermore, comparing the results with those of the stochastic model with internal noise (Equation (9)), the peak of the correlation value ρ is sharper, and the bell-shaped curve is more distinct. On the other hand, in the model without internal noise, when the input signal is large or the DC bias value is small, the correlation value ρ is monotonically decreasing with the application of noise. Specifically, for the condition that $V_{\mathrm{signal}}=$ 8 V or $V_{\mathrm{signal}}=$ 2 V and $V_{\mathrm{bias}}\leq$ 3 V, the input signal exceeds the threshold ($V_{\mathrm{th}}=9.4\times 10^{-1}$ V) even without external noise. However, in a stochastic model with internal noise, a gradual increase in the correlation value ρ due to external noise, i.e., a slight stochastic resonance effect, can be observed (Figure 16). In this case, the overall value of the correlation ρ is small compared to the model without internal noise. Furthermore, as seen when $V_{\mathrm{signal}}=$ 2 V and $V_{\mathrm{bias}}\geq$ 2 V and $V_{\mathrm{signal}}=$ 8 and $V_{\mathrm{bias}}=$ 3 or 4 V, the value of ρ is insensitive to external noise intensity. In other words, this means that a system with internal noise will maintain a constant signaling performance, independent of the intensity of external noise. Therefire it can be said that a system with internal noise is robust to external noise. This robust effect is thought to have reduced the slope of the monotonic decrease in the input-output correlation value ρ, resulting also in the decrease in the magnitude of the peak. In a system with internal noise, in addition to the robustness described above, an increase in the correlation value ρ due to stochastic resonance is observed even when the input voltage is above the threshold. This behavior is not seen in systems without internal noise, such as when $V_{\mathrm{signal}}=$ 2 V and $V_{\mathrm{bias}}\leq$ 3 V. Whilst a number of studies on stochastic resonance have dealt with subthreshold signals [170], it has been confirmed that stochastic resonance occurs even for signals above the threshold in systems with parallel threshold response systems [51,52]. This is due to the fact that the dynamic range of the threshold response is widened by parallelization. On the other hand, Suzuki et al. found that the internal noise effectively raises the threshold, which creats the situation that stochastic resonance was observed even for large input signals. This suggests that stochastic resonance phenomena can be effective for signals above a threshold in a noisy environment, even if the system is not parallelized. As the model with internal noise is shown in Equation (9), the internal noise is additive noise, where internal noise intensity is independent with input signal intensity. Figure 17 shows the structure for the signal flow in the OFET circuit system.

The structual validity of this signal flow was derived from the fact that the output strength of the OFET system was small and so the internal noise in the circuit was more pronounced. Since it was confirmed that the internal noise was superimposed on the output signal when without applying an external noise (Figure 14), it was found that there was no correlation between the internal noise and the external noise.

## 7. Analysis of Internal Noise in Organic Field Effect Transistor Systems

In Section 6, we described observation of the stochastic resonance in OFETs and it was suggested that the internal noise was assumed to be Gaussian white noise. In this section, the validity of this and also origin of the internal noise is discussed.

### 7.1. Internal Noise in Stochastic Resonance Experiments

#### 7.1.1. Characteristics of Internal Noise

**Figure 18:**
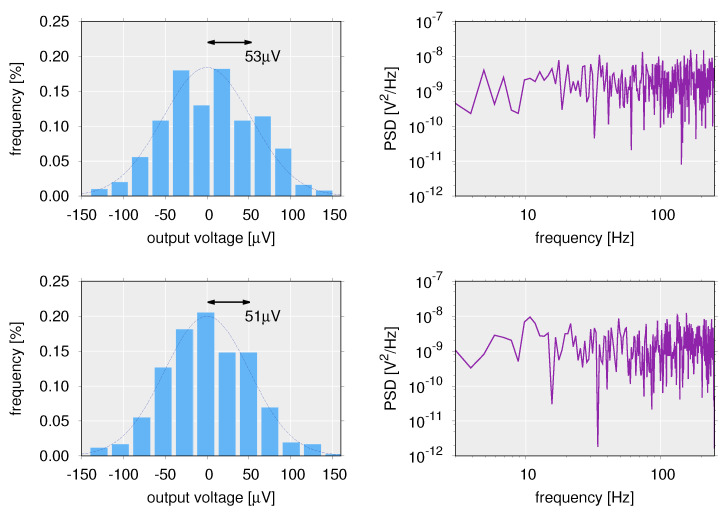
(**top left**) Distribution of output values below threshold for a drain−grounded circuit with the RR−P3HT−based OFETs and 4.4 kΩ. It is confirmed that they are distributed according to a normal distribution (dashed line) with variance (53 μV)${}^{2}$. (**top right**) Power spectral density of the output values below threshold for the drain−grounded circuit with the OFETs and 4.4 kΩ. A frequency−independent white spectrum is confirmed. (**bottom right**) Distribution of output values above threshold for a drain−grounded circuit with the OFETs and 4.4 kΩ. It is confirmed that they are distributed according to a normal distribution (dashed line) with variance (51 μV)${}^{2}$. (**bottom right**) Power spectral density of the output values above threshold for the drain−grounded circuit with OFETs and 4.4 kΩ. A frequency−independent white spectrum is confirmed [26]. Adapted with permission from *Applied Physics Letters* **2016**, *109*, 093702. Copyright 2016 American Institute of Physics.

In the measurement of the input-output characteristics of a drain-grounded circuit using an OFET, the frequency band for data recording was 5 kHz. The intensity distribution of the internal noise for the voltage region ($V_{\mathrm{in}}\geq 0$ V) below the threshold of the OFET system is shown in Figure 18 (top left) and the fast Fourier transform (FFT) spectrum is shown in Figure 18 (top right).

The result of this intensity distribution indicates that it is distributed according to a Gaussian distribution with standard deviation $5.3\times 10^{-5}$ V (Figure 18 (top left)). In addition, the power spectral density (PSD) is constant with respect to frequency. From this, it is found that the internal noise confirmed by the output below the threshold is Gaussian white noise. In addition, in order to analyze the internal noise above the threshold value, the switching output derived from the transfer characteristics of the OFET (Figure 14), i.e., the slow (low-frequency) fluctuation component, was removed using a Butterworth high-pass filter. The intensity distribution and spectrum of the filtered output values in the region above the threshold ($V_{\mathrm{in}}\leq -5$ V) are shown in Figure 18 (bottom left) and (bottom right), respectively. From Figure 18 (bottom left), it is confirmed that the output above the threshold is also Gaussian. The output values above the threshold of the OFET system processed by the filter and those in the subthreshold region were standard deviations of $5.1\times 10^{-5}$ V and $5.3\times 10^{-5}$ V, respectively. That is, regardless of whether the region exceeds the threshold or not, the internal noise intensity is constant and it has a Gaussian distribution. Furthermore, Figure 18 (bottom right) also shows a constant PSD regardless of frequency, indicating that the spectrum of the internal noise above the threshold is also a white spectrum independent of frequency. In summary, in the model of the OFET system used for the numerical simulations in Section 6 (Equation (9)), the internal noise is assumed to be a Gaussian white noise independent of the input, but this was also experimentally reasonable.

#### 7.1.2. Origin of Internal Noise

The noise observed in organic electronic devices includes thermal noise, shot noise, random telegraph noise, and 1/f noise [153] (Table 1). Thermal noise is generated as a result of the resistive component and has a white spectrum that is independent of frequency [171]. The shot noise increases in intensity with current value and shows a white spectrum [172,173]. Random telegraph noise is noise that transitions randomly between two or more discrete levels of voltage (current) and two levels of random telegraph noise show a Lorentzian spectrum [171,174,175,176]. The 1/f noise, as the name suggests, takes a spectrum with a slope of $1/f^{\gamma }$ (1≤γ≤2) [160,171,177,178,179,180].

The comparison has been made for these noise characteristics with the internal noise observed in the RR-P3HT-based OFET system. In an OFET system, i.e., a drain-grounded circuit with an OFET and a load resistor, the current value flowing to the load resistance is controlled by the voltage applied to the OFET. In other words, since the current value inside the system changes depending on the applied voltage, it is considered that the noise intensity changes depending on the applied voltage if the shot noise is dominant in the RR-P3HT-based OFET system. However, neither the magnitude nor the spectrum of the observed internal noise varied with the input voltage. So, while the spectra match in terms of being white, since the properties are different in terms of voltage dependence, it can be said that the internal noise is not shot noise. In the case of random telegraph noise or 1/f noise, the spectrum has a frequency-dependent form and is not a white spectrum. From this point of view, it seems that the internal noise of the OFET system, which showed a white spectrum, is not random telegraph noise nor 1/f noise.

On the other hand, thermal noise has a white spectrum, is independent of the applied voltage, and the characteristic is similar to the observed internal noise. Therefore, the internal noise is considered to be derived from thermal noise. However, the magnitude of the thermal noise generated by the resistive component *R* is

(10) $$\Delta V=\sqrt{4k_{B}TR\Delta f},$$

where $k_{B}$, *T*, and Δf are Boltzmann constant, temperature, and frequency band, respectively [181]. The magnitude of the thermal noise is calculated from the value of the load resistance (4.4 kΩ) in the OFET circuit: $\Delta V=6.0\times 10^{-7}$ V. The noise intensity was very much smaller than the observed internal noise. Therefore, it can be said that the thermal noise from the load resistor was not seen. Therefore, Suzuki et al. focused on the capacitor by the gate dielectric film of OFET. It is known that the intensity of thermal noise ΔV in RC circuits can be expressed by the equation below [181].

(11) $$\Delta V=\sqrt{k_{B}T/C},$$

where $k_{B}$, *T*, and *C* are Boltzmann’s constant, temperature, and capacitance, respectively. Using Equation (11), the noise intensity due to the gate-to-source capacitance of the OFET (about 1.6 pF) is calculated as $5.1\times 10^{-5}$ V. Since this value is in close agreement with the noise intensity observed at the output of the RR-P3HT-based OFET system, the internal noise that contributed to the stochastic resonance measurement experiments was a thermal noise characterized by an (insulating) dielectric layer, which is composed of poly(methyl methacrylate)[PMMA].

### 7.2. Load Resistance and Internal Noise

**Figure 19:**
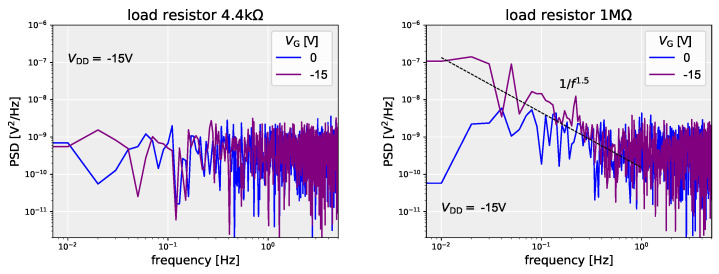
Power spectral density of the output values obtained when a constant voltage is applied to the drain-grounded circuit with the RR-P3HT-based OFET and 4.4 kΩ (**left**) and 1 MΩ (**right**). Regardless of the applied voltage, a frequency-independent white spectrum is observed [169]. Adapted with permission from doctoral dissertation of Yoshiharu Suzuki. Copyright 2019 Gunma University.

Here, we describe an investigation of what the internal noise would be like when the value of the load resistance was increased. For the load resistance in the drain ground circuit is 4.4 k and 1 MΩ, The noise spectra of the observed output voltages are Figure 19 (left) and Figure 19 (right), respectively, when $V_{G}$ and $V_{\mathrm{DD}}$ are constant. When the load resistance is 4.4 kΩ, a white spectrum can be seen regardless of the applied voltage. On the other hand, if the load resistance is increased to 1 MΩ, noise intensity decreases with increasing frequency when $V_{G}=-15$ V; that is, the 1/f spectrum was observed. It has been reported that OFETs show 1/f noise in $I_{\mathrm{DS}}$ [160,182,183]. It is thought that the 1/f current noise of OFET is converted to output voltage by the load resistance. When the load resistance is small, the current is output as a small voltage according to Ohm’s law. On the other hand, when the load resistance is large, the current noise is also converted to large voltages, resulting in output voltage with 1/f fluctuation. Also, the slope γ of the observed 1/f noise spectrum ($1/f^{\gamma }$) is γ=1.5. A value greater than unity means that application of the gate and drain voltages may cause an increase in the number of deep traps through which charge carriers should pass [162].

Let us now estimate the Hooge parameter $\alpha_{H}$ with respect to the observed 1/f noise. The Hooge parameter $\alpha_{H}$ is the relative noise intensity at 1 Hz, of the 1/f noise present in the system. First, the voltage noise observed from the value of the load resistance is converted to current. It is considered as the noise of the drain-source current of the OFET. Next, the relationship between power spectral density of current $S_{I}$ and Hooge parameter $\alpha_{H}$: [183,184]

(12) $$\frac{S_{I}}{I^{2}}=\frac{\alpha_{H}}{f^{\gamma }N_{C}},$$

is used, where *I* is the current value and $N_{C}$ is the total number of free charge carriers in the semiconductor. $N_{C}$ has the following form:

(13) $$N_{C}∼\frac{C_{i}WLV_{\mathrm{GS}}^{\mathrm{eff}}}{q}$$

calculated with the following parameters of $C_{i}$: capacitance of the insulating film per unit area, *W*: channel width, *L*: channel length, and *q*: elementary charge [183]. $V_{\mathrm{GS}}^{\mathrm{eff}}$ is the effective gate voltage and $V_{\mathrm{GS}}^{\mathrm{eff}}=V_{\mathrm{GS}}-V_{\mathrm{th}}$. From Equations (12) and (13),

(14) $$\begin{array}{ccc} \alpha_{H} & = & N_{c}\frac{S_{I}}{I^{2}} \end{array}$$

(15) $$\begin{array}{ccc} & ∼ & C_{i}WLV_{\mathrm{GS}}^{\mathrm{eff}}q\frac{S_{I}}{I^{2}} \end{array}$$

to calculate $\alpha_{H}$. The threshold voltage $V_{\mathrm{th}}$ of the OFET where the 1/f noise was observed is 0.90 V, and the Hooge parameter calculated from Equation (15) was $\alpha_{H}$∼2.6. This value is close to reported values ($\alpha_{H}$ = 4–50) of Hooge parameter for the bottom-gate-top-contact type OFET using P3HT [183] and also, in bottom-gate pentacene-based OFETs, $\alpha_{H}$ = 0.045–0.3 for top-contact type [158,185], and 5–20 for bottom contact type [185]. For the top-contact type with UV treatment of the insulating film, $\alpha_{H}$ = 1–3 [158]. From these it can be seen that the drain-to-source current of the OFET discussed in this review shows 1/f noise, which is comparable in magnitude to what has been observed so far.

## 8. Analysis of the Effect of Internal Noise on Stochastic Resonance

As we showed in Section 6 due to the internal noise controlled by the dielectric layer of the OFET, with the change in the intensity of the added external noise, it was confirmed that the variation of signal transmission performance was suppressed. Although the effect of signal enhancement by stochastic resonance phenomena is weakened, this robustness to external noise allows that the range of optimal external noise intensity in stochastic resonance is extended. This effect is similar to that seen in the parallel system [50].

The occurrence of stochastic resonance phenomena has been also discussed in a system without a threshold [186,187]. Even though there is no threshold in the nonlinear system, it has been shown that stochastic resonance can be enhanced by internal noise [186]. However, stochastic resonance in a single device element with internal noise had not been studied from the view point of signal-to-threshold distance. The signal-to-threshold distance is an important parameter governing the manifestation of stochastic resonance phenomena. Except for a nonlinear system based on a large number of parallelized networks [51] and the single bistable dynamic system [188] which has a synchronization lag, it was not possible to enhance a suprathreshold input signal due to external noise. Suzuki et al. have discussed the relationship between internal noise and stochastic resonance phenomena, especialy evaluation of noise robustness against external noise in a model considering the OFET characteristics, with focusing on the threshold of the nonlinear response system.

### 8.1. Theoretical Value of Parameter Normalization and Correlation Coefficient

In Section 6, the stochastic resonant OFET system was modeled as the nonlinear system (Equation (9)). In this model, the input voltage $V_{\mathrm{in}}\left(t\right)$, which is the sum of the input signal voltage R(t), the external noise voltage $\xi_{\mathrm{ext}}\left(t\right)$, and the DC bias $V_{\mathrm{bias}}$, is received by the nonlinear system with a threshold voltage $V_{\mathrm{th}}$ and an internal noise $\xi_{\mathrm{int}}\left(t\right)$. The input signal R(t) is a square wave and its intensity is $2V_{\mathrm{signal}}$, where each parameter of the model in Equation (9) is normalized and nondimensionalized by $V_{\mathrm{signal}}$ as follows.

(16) $$A^{\prime }=AV_{\mathrm{signal}}$$

(17) $$x\left(t\right)=R\left(t\right)/V_{\mathrm{signal}}$$

(18) $$\sigma_{\xi }\left(t\right)=\sigma_{\mathrm{ext}}\left(t\right)/V_{\mathrm{signal}}$$

(19) $$\theta =\left(V_{\mathrm{bias}}-V_{\mathrm{signal}}-V_{\mathrm{th}}\right)/V_{\mathrm{signal}}$$

(20) $$\sigma_{\eta }\left(t\right)=\sigma_{\mathrm{int}}\left(t\right)/V_{\mathrm{signal}},$$

where $\sigma_{\xi }$ and $\sigma_{\eta }$ are the intensities of the external noise ξ(t) and internal noise η(t) after the normalization, respectively, and satisfy the following conditions.

(21) $$\langle \xi \left(t\right)\xi \left(t^{\prime }\right)\rangle =\sigma_{\xi }^{2}\delta \left(t-t^{\prime }\right)$$

(22) $$\langle \eta \left(t\right)\eta \left(t^{\prime }\right)\rangle =\sigma_{\eta }^{2}\delta \left(t-t^{\prime }\right)$$

δ(t) is Dirac’s delta function. Here, *B* in Equation (9) was set as zero (B=0). The nonlinear response model after normalization is as shown in the following equation.

(23) $$y\left(t\right)=\left\{\begin{array}{cc} -A^{\prime }{\left(x\left(t\right)+\xi \left(t\right)-\theta \right)}^{2}+\theta \left(t\right) & (x(t)+\xi (t)<\theta ),\theta \\ \eta (t) & (\mathrm{otherwise}), \end{array}\right.$$

$A^{\prime }$ is a parameter that determines the steepness of the nonlinearity for the transfer characteristic of the OFET system. θ is the threshold and y(t) is the output of the system. In this model of p-type OFET, since the output of the drain-grounded circuit is not inverted with respect to the input, the On-state is reached when a negative voltage is input.

Since the input square wave voltage is also normalized by the input signal strength $V_{\mathrm{signal}}$, the input signal is a square wave that takes only binary values of 1 or −1, and the probability of each value is a duty ratio of *D* or 1−D. The random variables ξ and η, which are external and internal noise, respectively, are independent zero-mean Gaussian random variables, are assumed to satify stationary ergodicity, where the random variables have a time-independent probability distribution, where the ensemble mean is equal to the time mean. The cross-correlation coefficient ρ between the input signal *x* and the output signal *y* is a measure of signal transmission performance and can be described by the following equation.

(24) $$\rho =\frac{\mathrm{cov}[x,y]}{\sqrt{\mathrm{var}[x]}\;\mathrm{var}\left[y\right]},$$

cov[·] stands for covariance and var[·] for variance. When the external noise intensity $\sigma_{\xi }\neq 0$, the theoretical value of the correlation coefficient ρ in a nonlinear response system based on the OFET system is expressed as

(25) $$\begin{array}{cc} \rho = & \frac{-D\left(1-D\right)\left(E_{+}-E_{-}\right)}{\sqrt{D\left(1-D\right)\left(E_{2+}D+E_{2-}\left(1-D\right)+\frac{\sigma_{\eta }^{2}}{A^{\prime 2}\sigma_{\xi }^{4}}-{\left[E_{+}D+E_{-}\left(1-D\right)\right]}^{2}\right)}}, \end{array}$$

where

(26) $$\begin{array}{cc} E_{\pm }= & \frac{1}{\sqrt{2\pi \sigma_{\theta \pm }^{2}}}\exp\;\left(-\frac{1}{2\sigma_{\theta \pm }^{2}}\right)+\frac{1+\sigma_{\theta \pm }^{2}}{2\sigma_{\theta \pm }^{2}}\left[1+\mathrm{erf}\;\left(\frac{1}{\sqrt{2\sigma_{\theta \pm }^{2}}}\right)\right],\\ E_{2\pm }= & \frac{1+5\sigma_{\theta \pm }^{2}}{\sqrt{2\pi \sigma_{\theta \pm }^{6}}}\exp\;\left(-\frac{1}{2\sigma_{\theta \pm }^{2}}\right)+\frac{1+6\sigma_{\theta \pm }^{2}+3\sigma_{\theta \pm }^{4}}{2\sigma_{\theta \pm }^{4}}\left[1+\mathrm{erf}\;\left(\frac{1}{\sqrt{2\sigma_{\theta \pm }^{2}}}\right)\right], \end{array}$$

and

(27) $$\begin{array}{cc} \sigma_{\theta \pm }^{2}= & {\left(\frac{\sigma_{\xi }}{\mp 1+\theta }\right)}^{2}. \end{array}$$

Here erf is the error function [$\mathrm{erf}\left(z\right)=2\int_{0}^{z}\exp\left(t^{2}\right)\;dt/\sqrt{\pi }$]. $\sigma_{\theta \pm }^{2}$ is the variance value of the external noise normalized by the input signal-to-threshold distance. The correlation coefficient when the external noise intensity $\sigma_{\xi }=0$ is

(28) $$\begin{array}{ccc} \rho_{0} & = & \left\{\begin{array}{cc} 0 & (\theta \leq -1),\\ \frac{{\left(-1-\theta \right)}^{2}D\left(1-D\right)}{\sqrt{D\left(1-D\right)\left[{\left(-1-\theta \right)}^{4}\left(1-D\right)D+\frac{\sigma_{\eta }^{2}}{A^{\prime 2}}\right]}} & (-1<\theta \leq 1),\\ \frac{4\theta D(1-D)}{\sqrt{D\left(1-D\right)\left[16\theta^{2}\left(1-D\right)D+\frac{\sigma_{\eta }^{2}}{A^{\prime 2}}\right]}} & (1<\theta ). \end{array}\right. \end{array}$$

From Equations (25) and (28), the effect of internal noise intensity on the correlation coefficient is determined by $\sigma_{\eta }^{2}/{A\prime }^{2}=V_{\mathrm{int}}^{2}/{A\prime }^{2}$ and it can be seen that the smaller $A^{\prime }$ is, the larger the effect of internal noise is. This fitting parameter $A^{\prime }$ is related to the field-effect mobility of the FET and the larger the mobility is, the larger the $A^{\prime }$ is, but in most cases, OFETs are small compared to inorganic transistors (for the OFET system used in the literature, $A^{\prime }=3.0\times 10^{-6}$ V${}^{-1}$). Here we assume that the internal noise is white noise, but with comparison of the 1/*f* noise with the magnitude of the internal noise, organic transistors tend to have a large Hooge constant $\alpha_{H}$ compared to inorganic devices (For polymer FETs, $\alpha_{H}$∼4–50 [183], and for amorphous silicon thin-film transistors, $\alpha_{H}$∼$10^{-3}$ [189]). The Hooge constant $\alpha_{H}$ is the relative noise intensity at 1 Hz in 1/*f* noise [183]. Therefore, it can be said that the effect of internal noise is more expected in OFETs.

**Figure 20:**
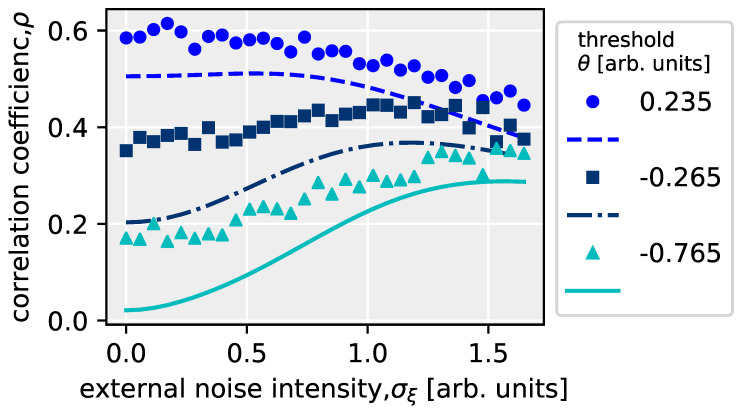
Variation of the correlation coefficient with respect to the external noise intensity at different thresholds. The points are the average of four experimental results obtained from the drain−grounded circuit with the RR−P3HT−based OFET and load resistance 4.4 kΩ. The curves represent the theoretical values calculated from Equations (25) and (28). In the experiments using the OFET system, the threshold θ is varied by changing the value of the input DC bias, which is normalized by the input signal strength ($V_{\mathrm{signal}}=4$ V) in the figure. The internal noise intensity is $\sigma_{\eta }=1.25\times 10^{-5}$ and the fitting parameter is $A^{\prime }=1.2\times 10^{-5}$ [27]. Adapted with permission from *Physical Review E* **2018**, *97*, 012217. Copyright 2018 American Physical Society.

With Equations (25) and (28) the correlation coefficient ρ is plotted as Figure 20 as a function of the external noise intensity $\sigma_{\xi }$. The experimental values obtained from the RR-P3HT-based OFET system are also drawn simultaneously. The threshold θ in the experiment is adjusted by changing the DC bias value applied to the OFET circuit. When the normalized threshold θ=−0.265,−0.765, the correlation coefficient increases with increasing external noise intensity, and the emergence of stochastic resonance phenomenon can be confirmed. At θ=0.235, the input signal is above the threshold, so the correlation coefficient ρ is monotonically decreasing. The analytical solution (theoretical value) can be in qualitative agreement with the experimental results. However, the correlation coefficients in the experiment are higher than the analytical values at all external noise intensities $\sigma_{\xi }$. This is the similar to the differences found in Section 6, between the experimental values (Figure 12) and the numerical simulations (Figure 16). The RR-P3HT-based OFETs have weak characteristics at high frequencies and the high-frequency components in the square wave input signal leak to the output, resulting in an output signal waveform that is more correlated with the input signal. This is the reason for the result that the experimental value was higher than the theoretical value.

Next, to evaluate the robustness to external noise, we use the derivative of the correlation coefficient (Equation (25)) with respect to the external noise intensity $\sigma_{\xi }$, we obtained the following equation.

(29) $$\begin{array}{c} \begin{array}{cc} \; & {\left|\frac{d\rho }{d\sigma_{\xi }}\right|}^{2}=\frac{D^{2}{\left(1-D\right)}^{2}}{\sigma_{\xi }^{2}}\;\{\frac{2A^{\prime }\sigma_{\xi }^{2}\left[\mathrm{erf}\;\left(\frac{1}{\sqrt{2\sigma_{\theta +}^{2}}}\right)-\mathrm{erf}\;\left(\frac{1}{\sqrt{2\sigma_{\theta -}^{2}}}\right)\right]}{{\left(\mathrm{var}\left[x\right]\;\mathrm{var}\left[y\right]\right)}^{1/2}}\\ \; & \;\;\;+\left[5-D\;\mathrm{erf}\;\left(\frac{1}{\sqrt{2\sigma_{\theta +}^{2}}}\right)-\left(1-D\right)\;\mathrm{erf}\;\left(\frac{1}{\sqrt{2\sigma_{\theta -}^{2}}}\right)\right]\\ \; & \;\;\;\times \left(\frac{4A^{\prime 2}\sigma_{\xi }^{4}\left[E_{+}D+E_{-}\left(1-D\right)\right]\;\mathrm{cov}[x,y]}{{\left(\mathrm{var}\left[x\right]\;\mathrm{var}\left[y\right]\right)}^{3/2}}\right){\}}^{2}, \end{array} \end{array}$$

The smaller the effect of external noise on the signal transmission performance of the system, the smaller the value of Equation (29) is.

### 8.2. Internal Noise and Threshold

**Figure 21:**
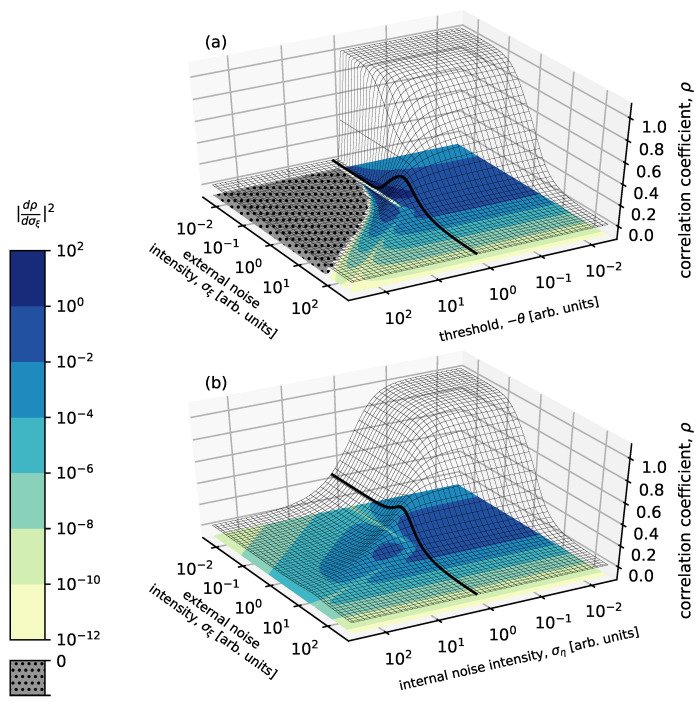
For the OFET model, the three-dimensional surfaces show the correlation coefficients and the contours show the square of the derivative of the correlation value with respect to the external noise intensity. (**a**) External noise intensity and threshold dependence, with internal noise intensity $\sigma_{\eta }=0$. and (**b**) external noise intensity and internal noise intensity dependence with a threshold θ=0. Here, the duty ratio of the input square wave is D=80% and the fitting parameter $A^{\prime }=1$ [27]. Adapted with permission from *Physical Review E* **2018**, *97*, 012217. Copyright 2018 American Physical Society.

As a function of the external noise intensity $\sigma_{\xi }$ and the threshold θ, the correlation coefficient ρ and the square of the derivative calculated using Equations (25) and (29), are plotted in Figure 21a. A bell-shaped curve of the correlation coefficient in the region where the input signal is below the threshold value (θ<−1) when the external noise intensity is a variable (e.g., the thick line in Figure 21a) is confirmed and the effect of stochastic resonance can be confirmed. On the other hand, in the region where the input signal is above the threshold (θ>−1) the correlation coefficient ρ decreases monotonically with external noise, and no stochastic resonance phenomenon is observed. When the external noise intensity is small (e.g., $\sigma_{\xi }\leq 10^{-1}$), the high system performance (ρ∼1) at θ>−1 suddenly drops to zero when the threshold becomes a condition below θ=−1.

If the threshold θ is replaced with the internal noise intensity $\sigma_{\eta }$, Figure 21b is obtained. The behavior is qualitatively similar to that for the threshold θ (Figure 21a). When the internal noise intensity is large (e.g., $\sigma_{\eta }\geq 1$), the correlation coefficient shows a bell-shaped dependence on the external noise intensity. Furthermore, high correlation coefficients ρ can be seen when $\sigma_{\xi }\leq 10^{-1}$ and $\sigma_{\eta }\leq 10^{-1}$. As the internal noise intensity is increased, it gradually drops to zero. Therefore, we can say that the internal noise intensity $\sigma_{\eta }$ has an effect similar to the threshold θ in nonlinear systems.

**Figure 22:**
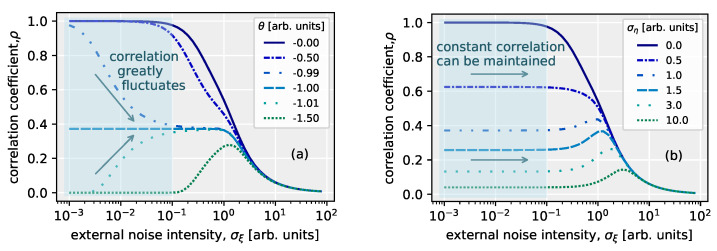
Variation of the correlation coefficient with respect to the external noise intensity in the OFET model. Variation of correlation coefficients at (**a**) different thresholds (when $\sigma_{\eta }=0$) and (**b**) different internal noise intensities (when θ=0). Here, the duty ratio D=80% and the fitting parameter $A^{\prime }=1$ [27]. Adapted with permission from *Physical Review E* **2018**, *97*, 012217. Copyright 2018 American Physical Society.

**Figure 23:**
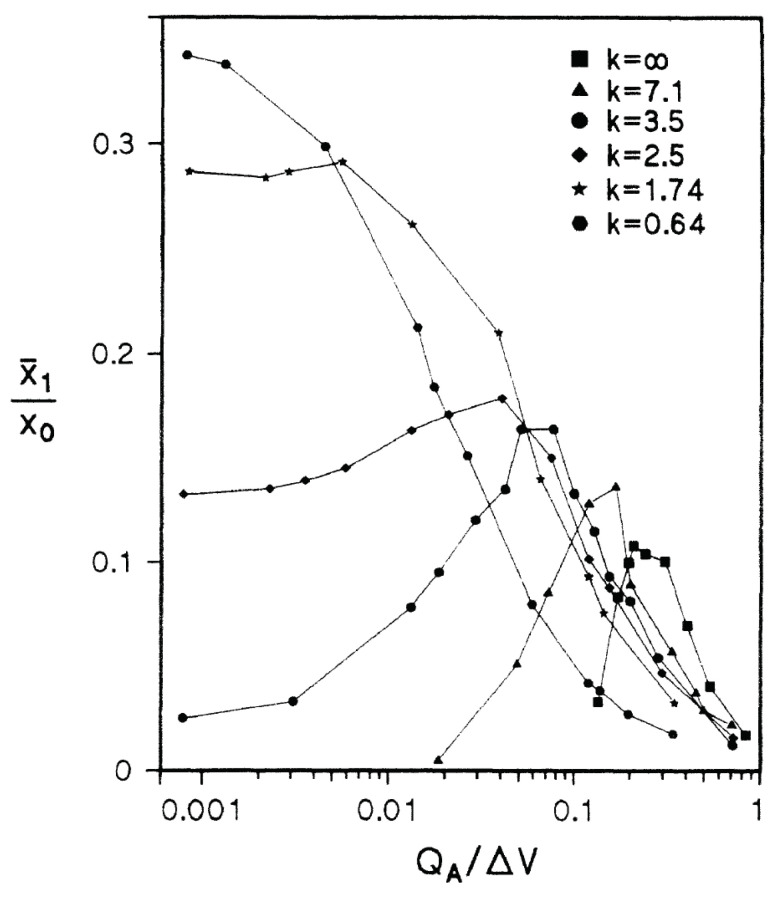
When additive and multiplicative noise are applied simultaneously along with periodic driving force in a bistable system. Output intensity $\bar{x_{1}}/x_{0}$ dependence on $Q_{A}$. $Q_{A}$ is the additive noise intensity and $Q_{M}$ is the multiplicative noise intensity, the latter of which satisfies $k=a/(2Q_{M})$. The potential barrier ΔV is determined by the parameters a,b. ($\Delta V=a^{2}/\left(4b\right)$): [190] adapted with permission from *Physical Review E* 1994, 49, 4878. Copyright 1994 American Physical Society.

However, when the value of the threshold θ changes around θ=−1, the behavior of the correlation coefficient with respect to the external noise changes markedly (Figure 22a). On the other hand, for various intensity of internal noise $\sigma_{\eta }$, the correlation value is almost independent with respect to the external noise intensity in the range $\sigma_{\xi }\leq 0.1$ (Figure 22b). In Figure 22b, which shows the system performance against external noise intensity, the behavior of the formation of plateau and peak shifts are observed by varying the internal noise intensity. These results are similar to those of a bistable system with multiplicative and additive noises, which is called multiplicative stochastic resonance [190]. While the internal noise has an effect of increase in the threshold, the threshold is moved in the opposite direction with an application of the multiplicative noise in Figure 23. But Both behaviors of changing the dependence of signal transmission on noise can be explained by the property of noise to change the threshold (the height of the potential barrier).

Looking at the square of the external noise intensity $\sigma_{\xi }$ derivative of the correlation coefficient ρ($|d\rho /d\sigma_{\xi }{|}^{2}$) in Figure 21a(color bar), a strong external noise dependence of the system performance (e.g., $|d\rho /d\sigma_{\xi }{|}^{2}>1$) is observed, but not in Figure 21b. Furthermore, in Figure 21b(color bar), the region $|d\rho /d\sigma_{\xi }{|}^{2}=0$ disappears. This is due to the bell-shaped dependence of ρ on external noise, where the peak of the ρ vs. $\sigma_{\xi }$ curve is broader for the case of existence of internal noise. The implication of these results is that although the internal noise acts like a threshold in a nonlinear response system, the system with internal noise becomes less dependent on external noise compared to one with a normal threshold.

Based on the results so far, when the input signal is above a threshold, such as even under conditions where no stochastic resonance phenomena were observed, stochastic resonance should be observable by adding noise to the "output signal" from the OFET. The addition of noise to the output signal is equivalent to the addition of internal noise to the system. This is because the internal additive noise is independent of the input signal. The increase in the correlation coefficient could not be confirmed experimentally for an OFET with a threshold of θ=0.235, as shown in Figure 20. The output signal obtained from the experiment with the OFET at this time was handled and random numbers following a Gaussian distribution as noise were added together. The correlation coefficients between the output signal and the input signal with the addition of this random number were calculated. If the standard deviation of the random number added to the output signal is increased, it is confirmed that the correlation coefficient with respect to the external noise intensity changes from a negative slope to a positive slope. In other words, by adding noise to the system’s output signal, a stochastic resonance phenomenon was able to be generated. This is because the input signal above the threshold was changed to a signal below the threshold by adding a random number as an internal noise. Therefore, either the internal noise of the OFET or the noise from the measuring instrument of OFET, or both, can be used to generate stochastic resonance. This means that the stochastic resonance phenomenon is observable in normally-On type FET and a system without a threshold. Furthermore, the stochastic resonance phenomenon can be controlled without adjustment by DC bias. In general, OFETs are more sensitive to atmospheric effects, with a positive threshold shift (in the case of p-type FETs) and Off-current is likely to increase [191,192,193]. In systems that would not have such a threshold, or in nonlinear systems that are receiving inputs above the threshold, stochastic resonance can be generated by additive internal noise. This is consistent with the previous study [186]. Here, since noise added to the output of the nonlinear response system was treated as internal noise, it may be said that the internal noise can be controlled even after the output signal is measured. However, in other nonlinear systems such as globally or locally coupled arrays [194,195], where internal noise is added before the input of the device, it is difficult to control the internal noise after the final output signal is measured. In such cases, the internal noise is expected to have a different effect.

## 9. Summary

We have tried to review noise-driven information processing in biological systems, particularly shed light on various types of stochastic resonance phenomena in Section 2 and also present examples of progressive application of noise in electronic devices in Section 3. In particular, in Section 4 we gave attension to noise found in organic electronic devices, which could have close connections with their strong molecular dynamics. In Section 5, we explored to clarify ideal condition for emergence of stochastic resonance phenomenon and discussed suitability of OFET for the realization. In Section 6, observation of stochastic resonance phenomena in OFETs using RR-P3HT as a semiconductor was described. A gradual variation in the correlation coefficient ρ between the input and output signals was observed with variation in external noise intensity. This small variation in ρ can be attributed to the contribution of the internal noise in the system. The stochastic resonance phenomenon seen in the presence of additive internal noise keeps the correlation value almost constant. These results suggest that internal noise may play a beneficial role in nonlinear responding systems. Athough internal noise exists in biological nervous system and it has been suggested that the use of stochastic resonance, internal noise could play a positive role in sudden environmental changes if the similar robust mechanism also effects in biological systems. Furthermore, even in the case of OFETs with weak nonlinearity, where stochastic resonance phenomenon is not clearly observed, the transfer characteristics of OFETs may play a significant role in the expression of stochastic resonance.

In Section 7, the internal noise of the OFET system was analyzed, and the Gaussian white noise was independent of the input voltage in the drain-grounded circuit. Therefore, it can be said that the treatment of internal noise in the numerical simulation in Section 6 was reasonable. From the characteristics of the internal noise, it is considered that the internal noise in the OFET system is the thermal noise controlled by the insulator (dielectric) layer of the OFET. However, in OFETs, which are semiconductor devices, it is said that 1/f noise can be observed. The reason why the 1/f noise did not act on the stochastic resonance experiment of interest is because load resistance is small with comparison to resistance of OFET. The existence of the 1/f noise in the RR-P3HT-based OFET system was confirmed by increasing the load resistance. However, since the frequency response of the system decreases as the load resistance increases, further improvement of the OFET characteristics will be required in order to make the 1/f noise act on the stochastic resonance phenomenon.

In Section 8, the OFET model was constructed and the effect of internal noise instead of the threshold for stochastic resonance was reviewed. By exploiting the additive internal noise, without adjusting the threshold or the applied DC bias, the input signal was able to be put into be subthreshold condition. Even when no stochastic resonance effect was experimentally observed because the input signal was suprathreshold, the stochastic resonance phenomenon was confirmed by adding noise to the output signal. From this result, it can be said that the effect is equivalent to the presence of internal noise even from outside the device. Also, from a comparison between the internal noise system and the threshold system, it is found that the former is more robust to external noise.

## 10. Future Perspectives

In this review, we have described observation of the stochastic resonance phenomenon using OFETs, which are devices with nonlinear response characteristics that can be extended to network devices because of their one-directional signal transmission. In the system treated in this review, the internal noise functioned as a threshold, but depending on the location where the internal noise acts, it may be used directly for the emergence of stochastic resonance phenomena. When the internal noise is added to the signal before the nonlinear response, it is used to induce stochastic resonance because the internal noise enters the location corresponding to the external noise in this case. The stochastic resonance phenomenon is a phenomenon in which the applied noise causes the input signal to cross the threshold, or it corresponds to the threshold being lowered by the noise. In other words, the threshold is lowered if the internal noise is applied before the threshold response, and raised if it is applied after. However, even if the internal noise is applied after the threshold response, if the system has a threshold response after the threshold response, such as a feedback system in which the output is re-input, or a feed-forward system in which the output is input to another nonlinear response system, the noise will be added before the threshold response, and therefore may contribute to emergence of the stochastic resonance. Therefore, even in this OFET system, the role of the internal noise may be different again when the system is networked.

On the other hand, the OFET system treated in this review has a problem in networking. That is, the output value of the signal is small. The channel resistance of the fabricated OFET is like to be a few MΩ even if the field effect is increased. Furthermore, the load resistance of the drain-grounded circuit is much smaller than that value, so the output signal could be very small, and the input signal attenuation might be severe. If the load resistance is made larger, the frequency characteristics of the OFET system will deteriorate, and the stochastic resonance phenomenon is likely to become less effective in the system. In other words, in order to network the system, the output signal of the OFET system must be increased and the frequency characteristics must be improved. One way to achieve this is to reduce the channel length of the OFET. The internal noise of the OFET system was white noise independent of the applied voltage, but it may be possible to fabricate a system with semiconductor-derived 1/f noise. As shown in the results of Section 7, it is possible to make a system with 1/f noise by increasing the load resistance of the drain-ground circuit. However, in order to make the stochastic resonance phenomenon occur, it is necessary to improve the frequency characteristics of the OFET, as already mentioned. Another idea is to change the semiconductor material used for the OFET. RR-P3DT, which is an organic semiconductor with a different length of alkyl side chains from RR-P3HT, has been observed to have relatively large conduction fluctuation, and it may cause large 1/f noise when used in OFETs. On the other hand, it has been reported that the mobility of OFETs using P3DT becomes smaller, so it is necessary to improve the OFET characteristics. The system with internal 1/f noise depending on the input voltage is expected to show more complicated stochastic resonance behavior because the noise is reflected in the signal transmission in a multiplicative manner and its variation varies with the time scale.

Here, we consider the use of stochastic resonance phenomena in electronic devices. The signal transmission performance achieved by the stochastic resonance phenomenon, as confirmed by the experiments and numerical calculations described in this review, is not very high, and the enhancement of the stochastic resonance effect cannot be expected to be much in a static system even if a parallelized network is fabricated as in the Collins model. Therefore, if the goal is to achieve high performance with noise or to amplify signals using stochastic resonance phenomena, it is necessary to deal with dynamic systems such as Schmitt triggers, which exhibit hysteresis characteristics with two thresholds. The stochastic resonance phenomenon is more effective in dynamic systems than in static systems. As for the amount of increase in signal transmission performance due to stochastic resonance phenomena, it can be said that inorganic transistors are larger than OFETs. This is shown by the fact that in Section 8, between the system with a dominant threshold response included in the nonlinear response characteristics and the system using internal noise as the threshold, a sharper stochastic resonant bell-shaped curve was observed in the former. However, OFETs, which have less nonlinearity than inorganic transistors and tend to have relatively large internal noise, do not perform as well but are more robust to external noise. Indeed, in contrast to the threshold of nonlinear response characteristics, whose value can significantly change the behavior of the system, internal noise does not change the behavior of the system much even if its intensity changes. The stochastic resonance phenomenon is a phenomenon that can be resolved to some extent by noise when the input signal is below the threshold, i.e., when there is a mismatch between the input signal and the detection range of the system. That is, a system with a robust threshold due to internal noise is less likely to create such a mismatch. In other words, the OFET system is expected to be a system that allows some degree of signal transmission without fine tuning of parameters such as the threshold and DC bias by using internal noise and stochastic resonance phenomena. In this aspect, OFETs are expected to be useful.

## Figures and Tables

**Table 1:** Classes of electrical noise observed in organic electronic devices. ($S_{I}$: current noise power spectral density, $k_{B}$: Boltzmann’s constant, *T*: Temperature, *R*: resistance, *q*: charge, *F*: Phono Factor, *I*: electric current, $\tau_{0}$: Relaxation time, *f*: frequency, and γ: exponent).

Noise Type	Power Spectral Density
Thermal noise	$S_{I}=\frac{4k_{B}T}{R}$
Shot noise	$S_{I}=2qF\left|I\right|$
Random telegraph noise	$S_{I}\propto \frac{1}{1+{\left(2\pi f\tau_{0}\right)}^{2}}$
1/f noise	$S_{I}\propto \frac{1}{f^{\gamma }}$

## Data Availability

The data that support the findings of this study are available on request from the corresponding author.
